# Engineering the synthetic β-alanine pathway in *Komagataella phaffii* for conversion of methanol into 3-hydroxypropionic acid

**DOI:** 10.1186/s12934-023-02241-9

**Published:** 2023-11-17

**Authors:** Sílvia Àvila-Cabré, Míriam Pérez-Trujillo, Joan Albiol, Pau Ferrer

**Affiliations:** 1 Department of Chemical, Biological and Environmental Engineering, Universitat Autònoma de Barcelona, Bellaterra, Catalonia Spain; 2 Servei de Ressonància Magnètica Nuclear, Facultat de Ciències i Biociències, Universitat Autònoma de Barcelona, Bellaterra, Catalonia Spain

**Keywords:** 3-Hydroxypropionic acid, *Pichia pastoris*, *Komagataella phaffii*, Methanol, β-Alanine pathway, Metabolic engineering

## Abstract

**Background:**

Methanol is increasingly gaining attraction as renewable carbon source to produce specialty and commodity chemicals, as it can be generated from renewable sources such as carbon dioxide (CO_2_). In this context, native methylotrophs such as the yeast *Komagataella phaffii* (syn *Pichia pastoris*) are potentially attractive cell factories to produce a wide range of products from this highly reduced substrate. However, studies addressing the potential of this yeast to produce bulk chemicals from methanol are still scarce. 3-Hydroxypropionic acid (3-HP) is a platform chemical which can be converted into acrylic acid and other commodity chemicals and biopolymers. 3-HP can be naturally produced by several bacteria through different metabolic pathways.

**Results:**

In this study, production of 3-HP via the synthetic β-alanine pathway has been established in *K. phaffii* for the first time by expressing three heterologous genes, namely *panD* from *Tribolium castaneum*, *yhxA* from *Bacillus cereus*, and *ydfG* from *Escherichia coli* K-12. The expression of these key enzymes allowed a production of 1.0 g l^−1^ of 3-HP in small-scale cultivations using methanol as substrate. The addition of a second copy of the *panD* gene and selection of a weak promoter to drive expression of the *ydfG* gene in the PpCβ21 strain resulted in an additional increase in the final 3-HP titer (1.2 g l^−1^). The 3-HP-producing strains were further tested in fed-batch cultures. The best strain (PpCβ21) achieved a final 3-HP concentration of 21.4 g l^−1^ after 39 h of methanol feeding, a product yield of 0.15 g g^−1^, and a volumetric productivity of 0.48 g l^−1^ h^−1^. Further engineering of this strain aiming at increasing NADPH availability led to a 16% increase in the methanol consumption rate and 10% higher specific productivity compared to the reference strain PpCβ21.

**Conclusions:**

Our results show the potential of *K. phaffii* as platform cell factory to produce organic acids such as 3-HP from renewable one-carbon feedstocks, achieving the highest volumetric productivities reported so far for a 3-HP production process through the β-alanine pathway.

**Supplementary Information:**

The online version contains supplementary material available at 10.1186/s12934-023-02241-9.

## Background

Fossil fuels not only constitute the most important energy sources in our everyday life, but they are also critical to produce plastics, synthetic materials, and other chemical products. However, the accelerated depletion of fossil resources and its undeniable relationship with global warming are forcing us to identify alternative feedstocks. Moreover, the use of plant-derived sugars as carbon sources comes into conflict with food commodities.

Methanol is increasingly gaining competitiveness as a microbial non-food feedstock for the chemical industry and biomanufacturing. This one-carbon compound is broadly available, presently obtained from syn-gas. It can also be prepared through a catalytic conversion that oxidizes a natural gas (methane). Another renewable alternative to produce methanol includes the reduction of atmospheric CO_2_ with hydrogen, promoting a sustainable circular carbon economy [1]. In addition, this carbon source has a greater degree of reduction per C-mol compared to most sugars, leading to potential higher product yields, and thus is considered a promising building block for synthesizing biofuels, commodity chemicals and biopolymers [2].

*Komagataella phaffii* (also known as *Pichia pastoris*) is a methylotrophic yeast strain capable of assimilating methanol as a sole source of carbon and energy. This is possible due to the presence of strongly induced genes encoding for enzymes involved in methanol metabolism, which are mostly located in the peroxisomes. After its discovery in the late 1960s, *K. phaffii* was first used for biomass and single-cell protein production from methanol [3, 4]. Later, *K. phaffii* was developed as a system to produce heterologous proteins for both academic and industrial purposes. The availability of the strong and tightly regulated methanol inducible *AOX1* promoter paved the ground for the exploitation of this non-conventional yeast for heterologous protein production. Synthetic biology toolboxes such as Golden*Pi*CS [5] and CRIS*Pi* kit [6], together with the growing knowledge base of its metabolism, have led *K. phaffii* to stand out as a specialized chassis not only to produce recombinant proteins, but also to metabolically engineer this yeast to produce added value metabolites [7]. In this context, the deployment of technologies for renewable methanol generation makes it an attractive feedstock for chemicals production using methylotrophic microorganisms. Methanol possesses more available electrons per carbon atom (reduction degree) than most sugars, which implies that more reducing power is being generated, usually resulting in enhanced product yields of desired metabolites [2]. Over the past 10 years, several chemicals have been synthesized in *K. phaffii* using methanol as C-source, including carboxylic acids such as malic acid, d-lactic acid and 3-hydroxypropionic acid (3-HP) [8–10], polyketides [11–13], and fatty acids derivatives [14]. Recently, the production of amino acids (β-alanine) from this renewable feedstock has also been reported in *K. phaffii* for the first time [15]. This β-amino acid is a precursor for the biosynthesis of several nitrogen-containing chemicals, such as pantothenic acid (vitamin B_5_).

3-HP was identified as one of the top value-added chemicals from biomass by the US Department of Energy (DOE) in 2004, since it was considered a key building block for both commodity and specialty chemicals production. 3-HP can be converted into 1,3-propanediol (PDO), acrylamide, acrylic acid, and methyl acrylate [16]. There is a growing demand for acrylic acid-based polymers used in the personal care industry, coatings, adhesives, and others. The global market size of this 3-HP derivative is expected to reach $19.2 billion by 2030 [17]. The bioconversion of renewable methanol into this three-carbon acid building block will allow us to reduce the unsustainable use of fossil resources, meaning less environmental pollution [18]. Furthermore, since *K. phaffii* is considered an acid-tolerant host, the fermentation process could be performed at a pH below the pKa value of 3-HP, obtaining the undissociated form of the acid, therefore reducing the costs of the downstream process [19].

Several microorganisms, both prokaryotes and eukaryotes, are 3-HP natural producers, although none of them produces a significant amount of this organic acid [20]. Three predominant heterotrophic routes named according to its precursor have been reconstructed into the industrial workhorses *Saccharomyces cerevisiae* and *Escherichia coli* for 3-HP synthesis. The highest 3-HP titer and overall process productivity were obtained with the coenzyme B_12_-dependent glycerol pathway implemented in *E. coli* [21]. Nevertheless, *E. coli* is not able to produce vitamin B_12_ on its own. Therefore, this expensive cofactor must be added to the culture medium, making the process economically unfeasible at an industrial scale [22].

Alternatively, the gene encoding for a bifunctional malonyl-CoA reductase from *Chloroflexus aurantiacus* (MCR_Ca_) has been expressed to implement the malonyl-CoA pathway in several model organisms, such as *E. coli* [23–26], *S. cerevisiae* [27–29] and *Schizosaccharomyces pombe* [30, 31], allowing for the production of 3-HP. Recently, we have successfully introduced the malonyl-CoA pathway in *K. phaffii* to produce 3-HP from glycerol [32]. After further metabolic engineering, the best strain produced up to 37.1 g l^−1^ of 3-HP at 0.71 g l^−1^ h^−1^ with a final product yield of 0.19 g g^−1^ in fed-batch cultures, resulting in the highest volumetric productivity reported so far in yeast [33]. Notably, a free fatty acids (FFA) overproducing *S. cerevisiae* strain engineered with the malonyl-CoA pathway has been recently optimized for an enhanced supply of metabolic precursors (malonyl-CoA and acetyl-CoA) and cofactor (NADPH), enabling the highest 3-HP production (56.5 g l^−1^) and product yield on glucose (0.31 g g^−1^) in fed-batch cultivations reported so far in yeast [34]. Similarly, the malonyl-CoA pathway has also been implemented in a FFA-overproducing *K. phaffii* strain to efficiently produce 3-HP from sole methanol, achieving a final titer of 48.2 g l^−1^, which is the highest reported 3-HP production from one-carbon feedstocks, and a product yield of 0.23 g g^−1^ [10].

In addition, 3-HP has been produced through the synthetic β-alanine pathway in *E. coli* [35, 36] and *S. cerevisiae* [28, 37]. This metabolic pathway consists of two steps, the first one converting β-alanine into malonyl semialdehyde (MSA) by either a β-alanine-pyruvate aminotransferase (BAPAT) or a γ-aminobutyrate transaminase (GABT), and the second one consisting in a reduction of MSA to 3-HP consuming either NADH or NADPH [38–40]. Several genes were tested for each one of these two steps to implement the pathway in *S. cerevisiae*, finally determining the expression of a BAPAT from *Bacillus cereus* (BAPAT_Bc_), along with a 3-hydroxypropionate dehydrogenase from *E. coli* K-12 (YDFG_Ec_), as the best option. Moreover, the endogenous biosynthesis of β-alanine is only active via spermine and has a very low flux. For this reason, an aspartate-1-decarboxylase from *Tribolium castaneum* (PAND_Tc_), catalyzing the conversion of L-aspartate into β-alanine, was expressed in *S. cerevisiae*. After further metabolic engineering of this strain, a final titer of 13.7 g l^−1^ of 3-HP with a 0.14 g g^−1^ product yield was obtained in a glucose-limited controlled fed-batch cultivation [37]. Recently, 3-HP production through the β-alanine pathway has been further optimized by applying phosphate-limiting conditions to a glucose-based fed-batch system, achieving a final 3-HP concentration of approximately 27 g l^−1^ with a product yield of 0.26 g g^−1^ [41].

In the present study, the enzymes used by Borodina et al. in *S. cerevisiae* [37] were expressed for the first time in *K. phaffii* to enable 3-HP production from methanol via the β-alanine pathway in our base strains. These base strains were further modified through cofactor engineering by overexpressing a *Pseudomonas* sp. 101 NADP^+^-dependent formate dehydrogenase variant. Fed-batch cultivations using methanol as the only carbon source in the feed were performed to evaluate the potential of this cell factory to generate an economically important product from this C1 feedstock.

## Results and discussion

### In silico comparison of the malonyl-CoA and the β-alanine pathways in *K. phaffii*

**Fig. 1:**
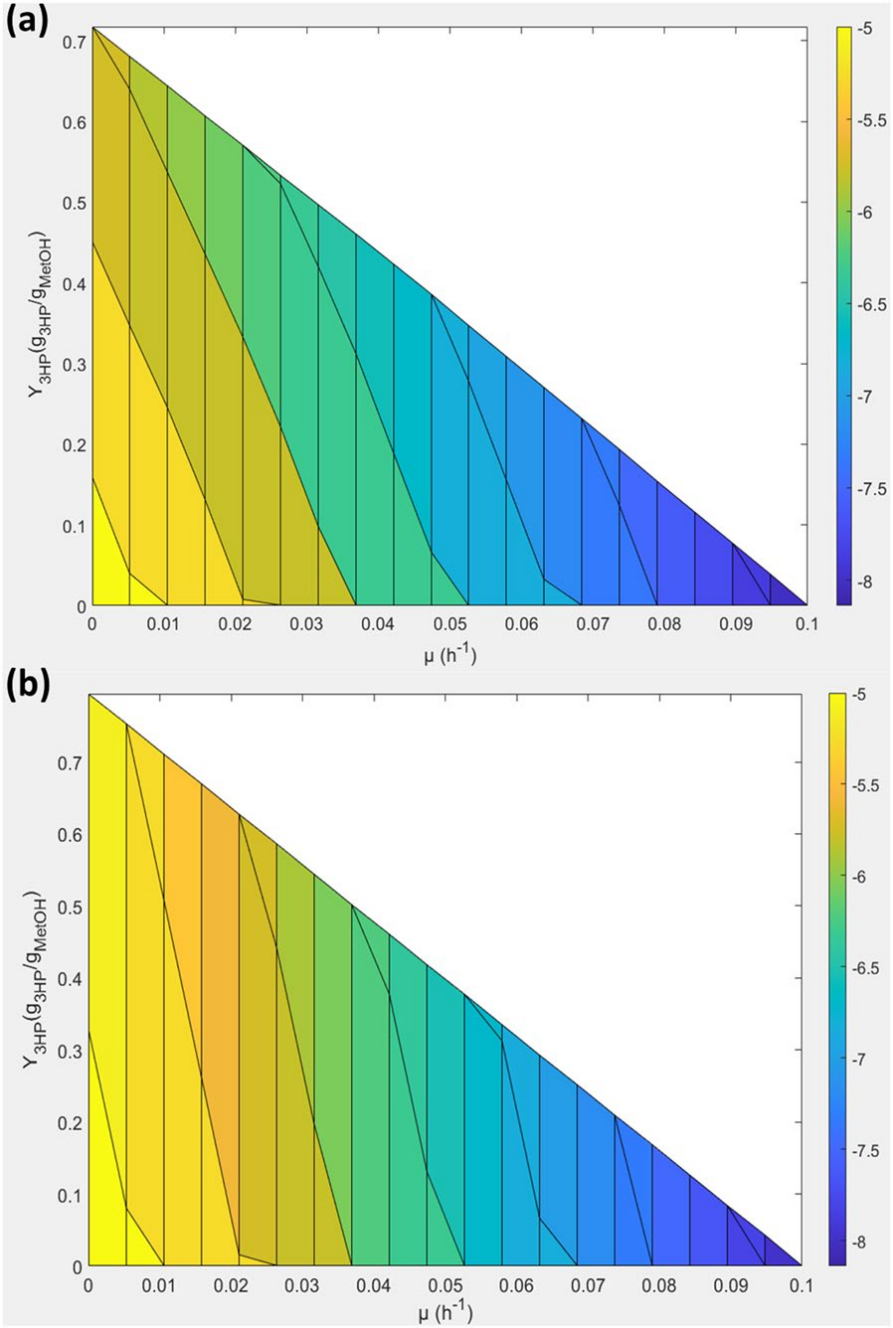
Quantitative prediction of metabolic capabilities of *K. phaffii* for 3-HP production from methanol. **a** Through the malonyl-CoA pathway. **b** Through the β-alanine pathway. A range of values for the specific growth rate was set, while limiting the system to different oxygen availability conditions. The 3-HP production was defined as the objective function to be maximized, obtaining the above phase planes after calculating the corresponding product yield for each predicted 3-HP production value. The color scale code indicates the specific oxygen uptake rate (q_O2_), from 5 (yellow) to maximal value set at 8 mmol O_2_ g_DCW_^−1^ h^−1^ (dark blue). Y-axis represents the product yield (Υ_3-HP_) (g_3-HP_ g_MetOH_^−1^), and the X-axis illustrates the growth rate (µ) (h^−1^)

The *K. phaffii*’s iMT1026 v3.0 genome-scale metabolic model [42] was used to evaluate the maximum 3-HP theoretical yield (Υ_3-HP max_) that could be achieved using methanol as sole carbon source through the malonyl-CoA and the β-alanine pathways. Assuming that all the carbon source was used for product synthesis, that is, not used for biomass growth (µ = 0 h^−1^), the maximum Υ_3−HP_ through the malonyl-CoA pathway was calculated to be 0.718 g_3-HP_ g_MetOH_^−1^ (Fig. 1a), whereas a higher value was obtained when using the β-alanine route (0.795) (Fig. 1b), mainly owing to a lower ATP requirement to produce one molecule of 3-HP from 3 molecules of methanol through the β-alanine pathway (see Additional file 1), resulting in slightly lower oxygen requirements compared to the malonyl-CoA pathway (Fig. 1a, b). The higher ATP requirement for 3-HP production through the malonyl-CoA pathway is mainly due to acetyl-CoA synthesis, which not only has an impact on the Υ_3-HP max_ value, but also results in higher oxygen dependence, as previously pointed out by Borodina et al. [37]. Overall, the β-alanine route stands out as a more attractive metabolic pathway towards 3-HP production at industrial scale.

### The expression of *panD*, *yhxA* and *ydfG* genes in *K. phaffii* generates a 3-HP-producing strain

**Fig. 2:**
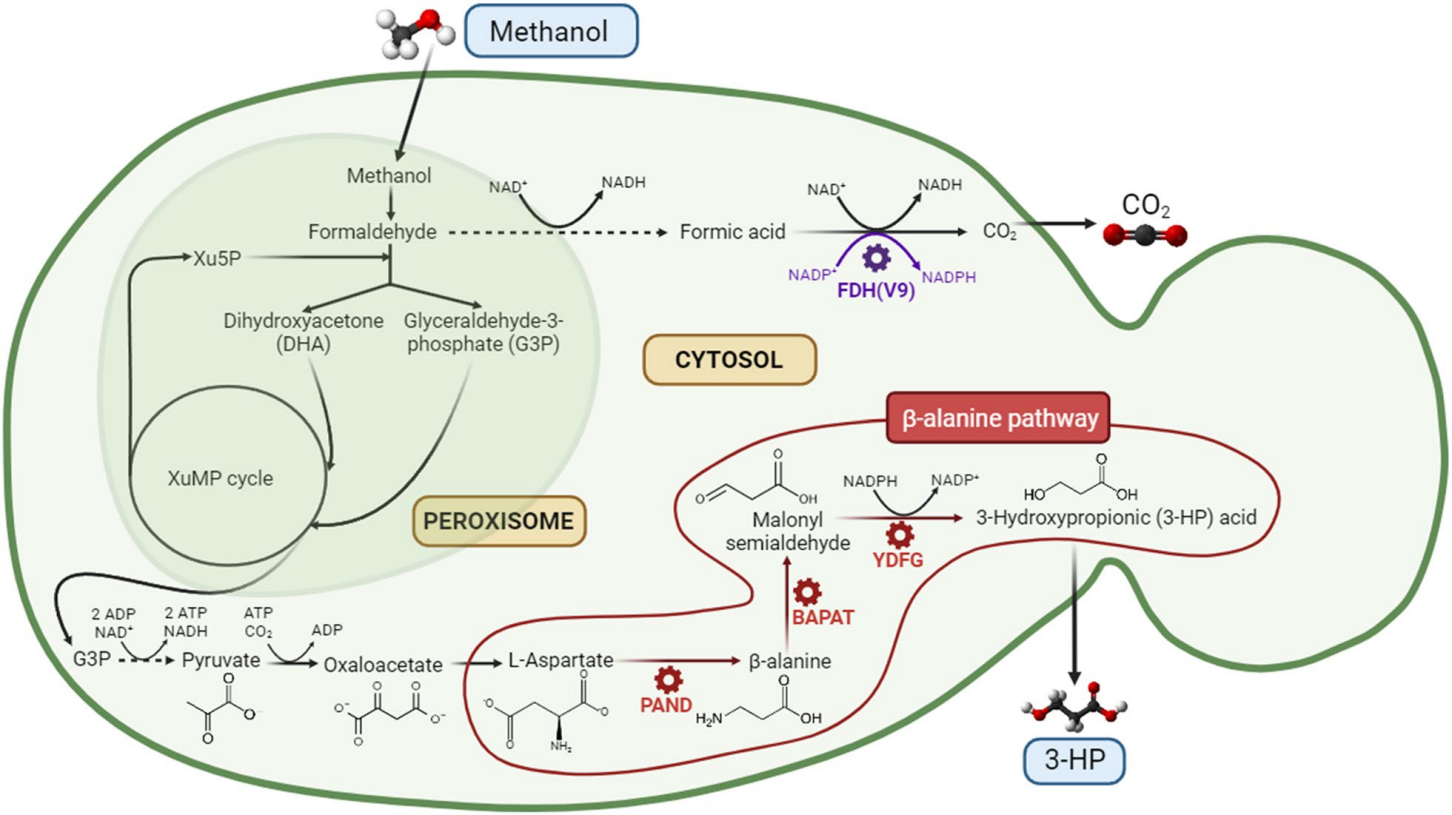
Schematic representation of the biological β-alanine route towards 3-HP production using methanol as substrate. The enzyme abbreviations are as follows: PAND – aspartate-1-decarboxylase, BAPAT – β-alanine-pyruvate aminotransferase, YDFG – 3-hydroxypropionate dehydrogenase, FDH(V9) – mutated formate dehydrogenase. The three heterologous enzymes required for the expression of the synthetic β-alanine pathway in *K. phaffii* CBS7435 parental strain are highlighted in red. The NADPH regeneration by FDH(V9) is shaded in purple

To reconstruct the synthetic β-alanine pathway for 3-HP production, the three codon-optimized *panD*, *yhxA* and *ydfG* genes encoding for a PAND from *T. castaneum*, a BAPAT from *B. cereus* and a YDFG from *E. coli*, respectively, were introduced into *K. phaffii* CBS7435, the parental strain. The strong and methanol inducible alcohol oxidase 1 (*AOX1*) and formate dehydrogenase (*FDH1*) promoters, were selected to conduct the expression of *panD* and *yhxA* genes, respectively. According to previous studies [37], the utilization of strong promoters such as the translation elongation factor EF-1 alpha promoter (P_*TEF1-α*_) for the expression of both *panD* and *yhxA* genes proved to be beneficial for 3-HP production with an engineered *S. cerevisiae* strain growing on glucose, since the reaction catalyzed by the PAND_Tc_ was determined to be the main flux control step of the β-alanine pathway. In these two first steps, an l-aspartate is transformed into β-alanine by the PAND_Tc_, which is in turn converted into MSA by the BAPAT_Bc_ enzyme. 3-HP is finally obtained through the reduction of MSA by the action of the YDFG_Ec_ (Fig. 2). Due to the NADPH-dependence of the latter enzyme, we expected the potential generation of a redox imbalance by the last step of the pathway. For this reason, instead of using a strong and methanol inducible promoter to control the expression of the *ydfG* gene, two different constitutive promoters showing a lower expression strength in comparison with the strong GAP promoter under methanol feed conditions (µ_max_ up to 0.1 h^−1^) were tested, namely the moderate strength mitochondrial porin (*POR1*) promoter, obtaining the PpCβ10 strain, and the weak pyruvate decarboxylase (*PDC1*) promoter, obtaining the PpCβ20 strain [5].

The PpCβ10 and PpCβ20 strains were grown up to an optical density at 600 nm (OD_600_) of 2.4 (see Additional file 2) on 24 deep-well plates with minimal medium containing 11.9 g l^−1^ (i.e., 1.5% v/v) of methanol, along with the reference strain (CBS7435). After 48 h of incubation, methanol was completely depleted. Since *K. phaffii* does not assimilate 3-HP as C-source [32], an endpoint sample was collected at the end of the culture to quantify the final 3-HP titers. The heterologous expression of *panD*, *yhxA* and *ydfG* genes resulted in 0.93 ± 0.03 g l^−1^ of 3-HP after 48 h of cultivation when the P_*POR1*_ was used to control the expression of the *ydfG* gene (PpCβ10 strain), whereas a slightly but significantly higher titer (p-value < 0.05), 1.04 ± 0.01 g l^−1^ 3-HP, was obtained with the weak P_*PDC1*_ driving expression of this gene (PpCβ20 strain). Notably, the 3-HP titers obtained herein were slightly higher than those achieved with a base *S. cerevisiae* strain expressing the same key enzymes under the control of strong constitutive promoters (*TEF1* and *PGK1*) and growing on a 20 g l^−1^ glucose medium (0.83 g l^−1^ 3-HP) [37]. Moreover, the expression of these three heterologous genes in *K. phaffii* resulted in similar 3-HP titers to those previously reported for a glucose-utilizing *S. cerevisiae* strain carrying overexpression cassettes for 5 genes, namely pyruvate carboxylase (*PYC1* and *PYC2*), BAPAT_Bc_, PAND_Tc_ and YDFG_Ec_. Only when xylose was used as substrate, a higher production of 3-HP was achieved (1.84 g l^−1^) with the latter strain [28]. These initial screening experiments pointed at *K. phaffii* as a promising chassis cell for the bioproduction of 3-HP from methanol.

### Improving 3-HP production by optimizing the flux through the β-alanine synthetic pathway

Once the functionality of the biosynthetic pathway was confirmed by the presence of 3-HP, we decided to improve the flux towards the production of this carboxylic acid by overexpressing the key biosynthetic enzymes. Previous studies with a *S. cerevisiae* strain expressing the β-alanine pathway, demonstrated that the effect of multiple integrations of PAND_Tc_ was larger than that of multiple copies of BAPAT_Bc_/YDFG_Ec_, thus suggesting that the major control point of the flux through the biosynthetic pathway were the *panD* gene transcriptional levels [37]. Accordingly, we hypothesized the overexpression of this gene as the most straightforward strategy for 3-HP improvement.

**Fig. 3:**
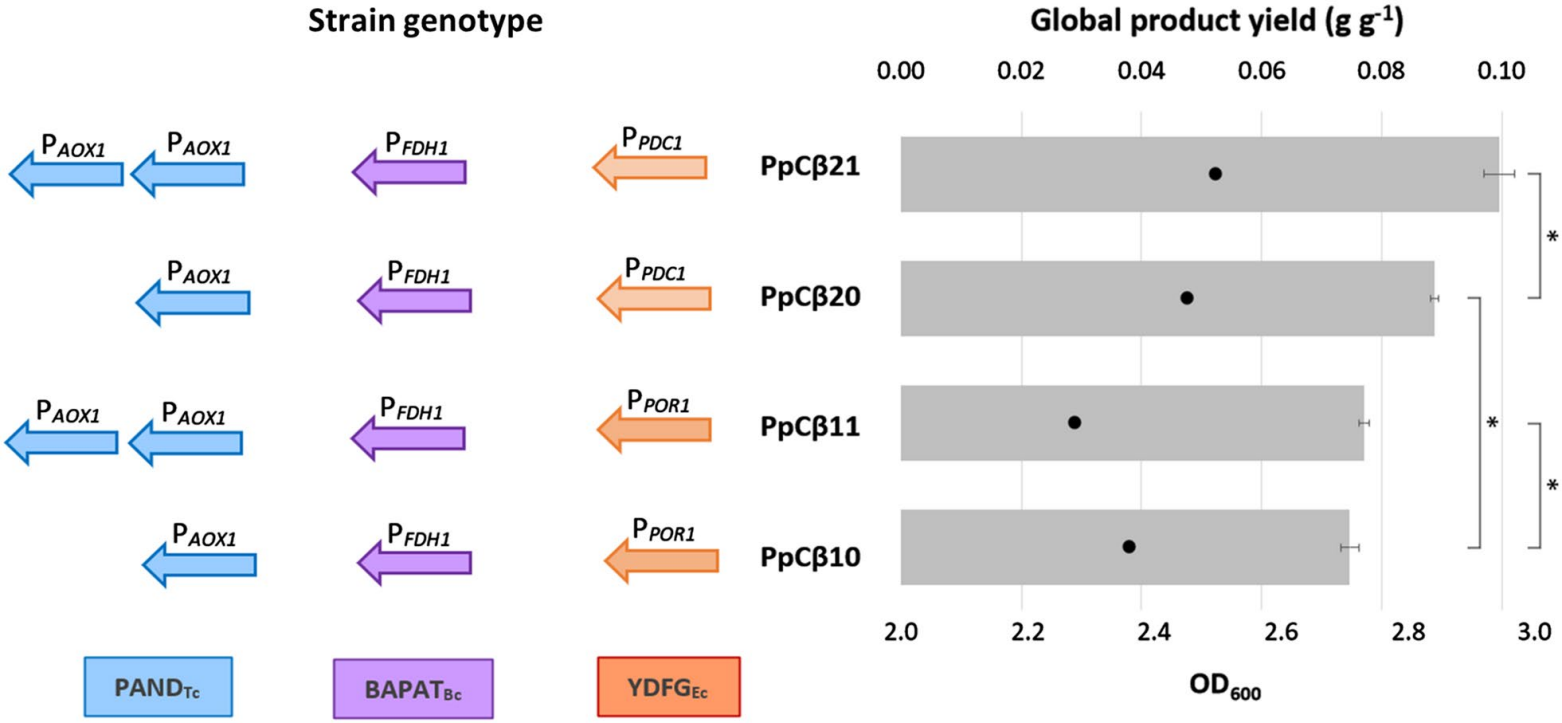
Global product yields (Y_P/S_) calculated for the base strains constructed in this study. The key heterologous enzymes expressed in each strain are depicted in the left side of the graph. One colored arrow corresponds to one copy of the related expression cassette (blue for PAND_Tc_, purple for BAPAT_Bc_, and orange for YDFG_Ec_). Promoters selected to drive expression of every transcriptional unit are mentioned above the arrows. On the right side, the grey bars show the average product yield (g g^−1^), and the black dots represent the averaged endpoint OD_600_. The error bars show the standard deviation, and the asterisk sign indicates a significant difference in global 3-HP yields between the strains indicated in brackets (p-values < 0.05)

The integration of a second copy of the PAND_Tc_ expression cassette into both PpCβ10 and PpCβ20 strains resulted in the PpCβ11 and PpCβ21 strains, respectively, which were cultivated on buffered minimal methanol medium (BMM) along with their parentals. After 48 h of cultivation, all strains reached a similar OD_600_ between 2.3 and 2.5 (see Additional file 2). The 3-HP production slightly increased by 3% (p-value = 0.04) in PpCβ11 strain (0.92 ± 0.01 g l^−1^ 3-HP), in comparison with its parental PpCβ10 strain (0.89 ± 0.02 g l^−1^), while the strain PpCβ21 reached the highest 3-HP concentration (1.18 ± 0.03 g l^−1^), i.e., a 12% increase respect to PpCβ20 (1.05 ± 0.01 g l^−1^). Notably, the 3-HP yield on methanol was increased by 11% with the additional copy of the *panD* gene (strain PpCβ21) while this effect was much lower in the PpCβ11 strain (3%) (Fig. 3). These results indicate that lower expression levels of the *ydfG* gene (i.e., under the control of P_*PDC1*_) seem to be beneficial in terms of 3-HP production. This suggested the presence of a bottleneck at the final step of the pathway, since the YDFG_Ec_ requires NADPH to catalyze the reduction of the malonic semialdehyde to 3-HP, which is not being supplied. We hypothesized that this perturbation of the NADPH homeostasis could be further exacerbated when the β-alanine flux into the pathway is increased (i.e., with the insertion of an additional copy of the *panD* gene under the very strong *AOX1* promoter), resulting in a potential limitation of NADPH equivalents for anabolism/cell growth.

### Engineering the redox balance by overexpressing the PseFDH(V9) enzyme variant from *Pseudomonas* sp. 101, a NADP^+^-dependent formate dehydrogenase

To increase NADPH supply, the gene coding for a mutated formate dehydrogenase from *Pseudomonas* sp. 101, namely PseFDH(V9), was expressed in both PpCβ20 and PpCβ21 strains, obtaining the PpCβ20-P and PpCβ21-P strains, respectively. The enzyme variant PseFDH(V9) shows the highest catalytic efficiency (k_cat_/K_M_) for NADP^+^ that has ever been reported for any FDH towards this cofactor, mainly attributed to a very low K_M_^NADP^ = 0.026 mM. This causes PseFDH(V9) to reduce NADP^+^ with barely any competition from NAD^+^, even though the high concentration of this competing substrate in the cytosol [43]. The gene encoding for the PseFDH(V9) enzyme was expressed under the control of the strong and methanol inducible *FDH1* promoter. The PpCβ20-P and PpCβ21-P strains were cultivated along with their corresponding parental strains on 24-deep well plates containing BMM. A comparable endpoint OD_600_ between 2.4 and 2.6 was reached after 48 h of cultivation by the strains PpCβ20, PpCβ21 and PpCβ20-P. Conversely, cell growth was slightly lower for the PpCβ21-P strain (DO_600_ = 2.2) (see Additional file 2). The final 3-HP titer was significantly reduced by about 11% in strain PpCβ20-P (1.12 ± 0.01 g l^−1^) compared to its parental strain PpCβ20 (1.24 ± 0.03 g l^−1^) (p-value < 0.05). The introduction of the PseFDH(V9) enzyme in strain PpCβ21 resulted in a minor but also significant decrease (p-value < 0.0003) of about 6% on the final product titer, from 1.29 ± 0.01 g l^−1^ to 1.22 ± 0.01 g l^−1^ (PpCβ21-P). However, there were no significant differences (p-value = 0.38) in the 3-HP produced per OD_600_ unit between these strains. In summary, the expression of this NADP^+^-dependent formate dehydrogenase did not improve neither 3-HP production nor product yields using methanol as sole carbon and energy source in small-scale experiments.

### Evaluating the impact of the β-alanine pathway on cell growth kinetics

To determine the maximum specific growth rate (µ_max_) of the different strains obtained in this study, series of growth kinetics experiments were performed by cultivating a representative clone from each strain in shake flask cultures using BMM medium. The introduction of the β-alanine route led to a reduction of the µ_max_ from 0.14 h^−1^ (reference CBS7435 strain) to 0.11 h^−1^ and 0.09 h^−1^ for PpCβ20 and PpCβ10 strains, respectively (see Additional file 2). The β-alanine pathway consumes 1 NADPH per 3-HP, thereby reducing NADPH availability for anabolic purposes, which has been previously correlated to decreased biomass yields on methanol [44]. Coherently, lower expression levels of the NADPH-consuming YDFG_Ec_ enzyme (i.e., under the control of P_*PDC1*_, strain PpCβ20) of the β-alanine pathway proved to be beneficial, both in terms of 3-HP production and cell growth. Moreover, this effect was more pronounced with the overexpression of the PAND_Tc_ expression cassette, as inferred from the µ_max_ comparison between strains PpCβ21 and PpCβ11 (see Additional file 2). Introducing a second *panD* gene copy into the PpCβ10 strain substantially decreased the µ_max_ value from 0.09 h^−1^ to 0.06 h^−1^ (PpCβ11), whereas this effect was minimal in the PpCβ20 strain, leading to a slight (not statistically significant, p-value = 0.28) reduction from 0.11 h^−1^ to 0.10 h^−1^ (PpCβ21). According to previous studies [15], fine-tuning the *panD* gene dosage aiming at increasing the flux towards the β-alanine intermediate through aspartate decarboxylation may cause an impaired cell growth of *K. phaffii*, since aspartate takes part in many biological processes. The µ_max_ value obtained for the PpCβ11 strain was consistent with this phenomenon, albeit we did not observe this effect in the PpCβ21 strain. Further metabolic flux analyses should help elucidating the cause of the differences between the strains PpCβ11 and PpCβ21 growth. In any case, PpCβ11 was discarded for further bioreactor-scale cultivation experiments.

The introduction of the mutated formate dehydrogenase FDH(V9) from *Pseudomonas* in the PpCβ20 strain did not affect cell growth on methanol, i.e., both PpCβ20 and PpCβ20-P strains showed the same µ_max_ (0.11 h^−1^). In contrast, the PpCβ21-P strain showed a lower µ_max_ (0.08 h^−1^) compared to its parental strain PpCβ21. Since both strains showed similar 3-HP production titers, further characterization in bioreactor-scale experiments was required.

### Production of 3-HP in fed-batch cultivations

Initial fed-batch cultivations of the 3-HP-producing strains using a growth defined mineral medium typically used for recombinant protein production in *K. phaffii* [45], which is only supplemented with biotin, revealed that such medium did not support neither growth nor 3-HP production during the feed phase on methanol. We hypothesized this was probably due to the special co-factor requirements described for some of the enzymes of the β-alanine pathway, specifically for the PAND_Tc_ (*UniProtKB*: *A7U8C7*) and BAPAT_Bc_ (*UniProtKB*: *C2VE79*) enzymes, which use at least one pyridoxal phosphate as cofactor for its synthesis according to the UniProtKB database [46]. This coenzyme is derived from pyridoxine (vitamin B_6_) and plays an important role in amino acid metabolism. Moreover, β-alanine is an intermediate in pantothenic acid (vitamin B_5_) biosynthesis, which, in turn, is an integral part of the coenzyme A and acyl carrier protein, co-factors required for several enzymes [37]. The redirection of this β-amino acid flux towards the production of 3-HP instead of vitamin B_5_, could be an explanation for the observed growth impairment in the bioreactor-scale cultivations. Indeed, the presence of both vitamins B_6_ and B_5_ in the yeast nitrogen based (YNB)-containing medium used in the small-scale cultivations is likely to be the reason why the 3-HP-producing strains grow normally in those conditions. Subsequently, bioreactor cultivations were carried out using a growth medium supplemented with a vitamin solution containing, among others: calcium pantothenate (B_5_), pyridoxine hydrochloride (B_6_), and niacin (B_3_), which is an important precursor for the essential redox cofactors NAD^+^ and NADP^+^. This vitamin solution is usually added to Delft medium, commonly employed for bioreactor cultivations of *S. cerevisiae* [47], allowing for sustained growth on methanol and 3-HP production.

The best performing strains PpCβ20 and PpCβ21, along with the strain expressing the mutated FDH(V9) enzyme from *Pseudomonas*, PpCβ21-P, were further evaluated in aerobic fed-batch reactors. After the initial glycerol batch and methanol adaptation (transition) phases, the fed-batch phase was started, where methanol was fed following a pre-programmed exponential feeding strategy for controlled growth rate at µ = 0.03 h^−1^. The feeding phase was finalized before reaching a dry cell weight (DCW) of 50 g l^−1^ of biomass, accounting for 39.3 h of the total process duration, with a total methanol concentration added into the culture of almost 125 g l^−1^. Neither methanol nor by-products were detected by HPLC analysis over the course of fermentation. Nonetheless, exometabolome profiling analyses by NMR of the supernatant samples from the early, mid, and late methanol-feeding phase revealed the presence of small amounts (mM range, below HPLC detection limit) of ethanol as well as some metabolites potentially derived from leucine (Leu) and isoleucine (Ile) biosynthesis and degradation pathways (or pyruvate fermentation) such as 2-oxoisocaproate and 3-methyl-2-oxopentanoate (see Additional file 3 for NMR spectra of the culture supernatants). Both compounds are involved in transamination reactions with 2-oxoglutarate and l-glutamate, which, in turn, are also present in the oxaloacetate conversion into l-aspartate catalyzed by an aspartate aminotransferase (*AAT2*), reaction that takes place upstream of the β-alanine pathway (see Fig. 2). 3-Hydroxyisobutyrate, pyruvate and acetate were also detected in much lower amounts as well as traces of methanol. In contrast to 3-HP-producing *K. phaffii* strains engineered with the malonyl-CoA pathway growing in fed-batch cultures using glycerol as C-source [32], no arabitol was accumulated during the fermentation process.

**Fig. 4:**
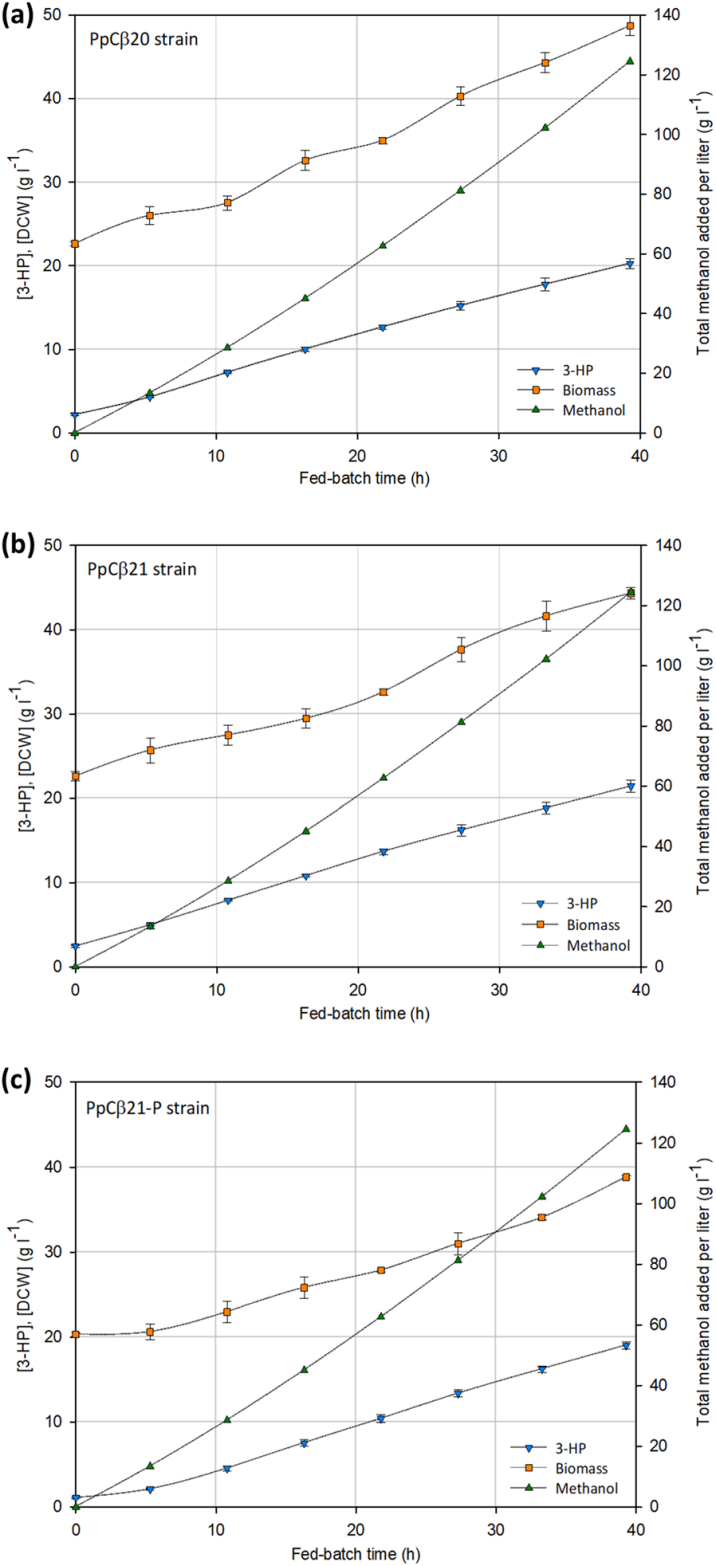
Fed-batch phase profiles from the bioreactor-scale experiments performed with the 3-HP-producer strains **a** PpCβ20, **b** PpCβ21 and, **c** PpCβ21-P. Concentration of dry cell weight and 3-HP are represented in the left-side y-axis. The total amount of methanol added, normalized by the actual volume of the reactor at every time, is represented using the right-side y-axis. The cultivation profile shown for each strain corresponds to the average result of two independent cultivations. The error bars denote the standard deviation for the duplicate

At the end of the culture, the PpCβ21 strain produced up to 21.4 ± 0.7 g l^−1^ of 3-HP, whereas 20.3 ± 0.6 g l^−1^ were achieved by the strain PpCβ20. In both cases the 3-HP generated during the batch phase of the cultures was slightly above 2 g l^−1^ (Fig. 4). The addition of a second copy of the *panD* gene in PpCβ21 resulted in a 12% improvement of the final 3-HP titer in the small-scale screenings. This increase was reduced to 6% when both strains were tested in bioreactor fed-batch cultivations. The 3-HP yield on biomass (Y_P/X_) calculated for the fed-batch phase was increased by 18% in PpCβ21 with respect to PpCβ20. Consequently, this resulted in a 10% improvement of the specific productivity (q_p_) (Table 1), and thus demonstrating that an enhanced supply of β-alanine intermediate in the metabolic pathway had a positive effect on 3-HP production, coherent with previous studies [37]. The expression of the PseFDH(V9) enzyme in PpCβ21-P strain allowed for a final 3-HP titer of 19.0 ± 0.5 g l^−1^, along with lower biomass concentration levels (38.8 g l^−1^ DCW) (Fig. 4), in comparison with the above-mentioned strains. This yielded a further 14% and 10% increase in Y_P/X_ and q_p_, respectively, while the product yield on methanol (Y_P/S_) remained unchanged for both PpCβ21-P and PpCβ21 (Table 1). Furthermore, the specific carbon dioxide evolution rate (q_CO2_) was significantly increased by 29% (p-value = 0.02) when comparing the PpCβ21-P to the PpCβ21 strain, whereas there were no significant differences (p-value = 0.10) in the q_CO2_ between strains PpCβ20 and PpCβ21 (Table 1 and Additional file 4). Consistently, the PpCβ21-P showed the highest CO_2_ yield on methanol (Y_CO2/S_) among the three strains (Table 1). Therefore, it is plausible that the introduction of the ectopic NADPH-regenerating reaction from formate causes a carbon flux redistribution through the methanol dissimilation pathway, that is, generating more CO_2_ while providing a higher supply of NADPH. This hypothesis is consistent with the higher Y_P/X_ and q_P_ values observed for the strain PpCβ21-P, as 3-HP synthesis requires a supply of reduction equivalents. In addition, the biomass yield (Y_X/S_) obtained for the PpCβ21-P strain was reduced compared to the other strains. Since the total amount of methanol consumed by all strains was the same, this was also reflected in a significant increase (p-value = 0.036, see Additional file 4) of the specific methanol consumption rate (q_s_) of the PpCβ21-P strain in comparison to the PpCβ21 strain (Table 1). Overall, it appears that overexpression of the heterologous PseFDH(V9) gene caused a carbon flux redistribution towards CO_2_ and 3-HP production to the detriment of biomass generation.

**Table 1:** Averaged value of key process parameters obtained for the methanol fed-batch phase using a pre-programmed µ of 0.03 h^−1^

	PpCβ20	PpCβ21	PpCβ21-P
Q_P_ (g_3-HP_ l^−1^ h^−1^)	0.46 ± 0.01	0.48 ± 0.02	0.46 ± 0.01
Y_X/S_ (g_DCW_ g_MetOH_^−1^)	0.28 ± 0.01	0.25 ± 0.01	0.21 ± 0.01
Y_P/S_ (g_3-HP_ g_MetOH_^−1^)	0.14 ± 0.01	0.15 ± 0.01	0.15 ± 0.01
Y_P/X_ (g_3-HP_ g_DCW_^−1^)	0.52 ± 0.03	0.61 ± 0.03	0.69 ± 0.03
Y_CO2/S_ (g_CO2_ g_MetOH_^−1^)	0.70 ± 0.02	0.73 ± 0.02	0.82 ± 0.03
q_S_ (g_MetOH_ g_DCW_^−1^ h^−1^)	0.103 ± 0.002	0.107 ± 0.002	0.124 ± 0.002
q_P_ (mmol_3-HP_ g_DCW_^−1^ h^−1^)	0.166 ± 0.001	0.183 ± 0.001	0.201 ± 0.001
q_CO2_ (mmol_CO2_ g_DCW_^−1^ h^−1^)	1.65 ± 0.01	1.79 ± 0.01	2.30 ± 0.01
µ (h^−1^)	0.029 ± 0.001	0.027 ± 0.001	0.026 ± 0.001

Volumetric productivity (Q_P_), biomass yield on methanol (Y_X/S_), 3-HP yield on methanol (Y_P/S_), 3-HP yield on biomass (Y_P/X_), CO_2_ yield on methanol (Y_CO2/S_), specific substrate consumption rate (q_S_), specific 3-HP production rate (q_P_), specific carbon dioxide evolution rate (q_CO2_), and experimentally measured mean specific growth rate (µ). Cultivations were performed in duplicate and biomass concentration analyses were performed in triplicate. ± indicates SD of the biological replicates

The iMT1026 v3.0 genome-scale metabolic model of *K. phaffii* [42] was used to validate the experimental 3-HP yields resulting from fed-batch cultivations. For the simulations, the experimental values obtained for the main macroscopic variables (i.e., q_S_, q_O2_, q_CO2_, and µ) of each methanol-grown strain were set as constraints, and q_3-HP_ was maximized. Y_P/S_ and Y_P/X_ were calculated for each strain using the predicted q_3-HP_ values (Table 2). Notably, the model showed reasonably accurate predictions, with overall deviations around 6–15% in most cases, even though the mean elemental biomass composition and the estimated non-growth associated maintenance energy (NGAME) parameter that were used as constraints to perform these simulations were taken from chemostat cultivations of a non-producing (wild-type phenotype) *K. phaffii* strain growing on methanol [42]. The use of condition-specific biomass composition equations and the recalibration of energetic parameters (i.e., NGAME and GAME) through experimental data derived from chemostat cultivations should allow for a greater accuracy in the prediction of physiological macroscopic parameters.

**Table 2:** Comparison of theoretical performance of iMT1026 v3.0 with experimental data from the methanol fed-batch cultivations at µ = 0.03 h^−1^

	PpCβ20	PpCβ21	PpCβ21-P
Predicted Y_P/S_	0.16	0.17	0.16
Experimental Y_P/S_	0.14 ± 0.01	0.15 ± 0.01	0.15 ± 0.01
Relative deviation (%)	6–19	6–18	0-12.5
Predicted Y_P/X_	0.57	0.68	0.77
Experimental Y_P/X_	0.52 ± 0.03	0.61 ± 0.03	0.69 ± 0.03
Relative deviation (%)	3.5–14	6–15	6.5–14

3-HP yield on methanol (Y_P/S_) (g_3-HP_ g_MetOH_^−1^), 3-HP yield on biomass (Y_P/X_) (g_3-HP_ g_DCW_^−1^). ± indicates SD of the biological replicates

Interestingly, the highest C-yield (C-mol of 3-HP per C-mol of substrate) calculated for the methanol feeding phase of fed-batch cultivations (0.16 ± 0.01 Cmol Cmol^−1^) is comparable to that achieved by a *S. cerevisiae* strain overexpressing *AAT2*, *PYC1*, *PYC2*, BAPAT_Bc_, YDFG_Ec_, and multiple copies of PAND_Tc_ growing on glucose (0.15 ± 0.0 Cmol Cmol^−1^, calculated from data provided in Borodina et al. [37]). The conversion of 3 molecules of methanol to one molecule of 3-HP through the β-alanine pathway is an ATP-consuming process, whereas it has no net ATP consumption when glucose is used as substrate (see Additional file 1: Table S1). Therefore, the Υ_3−HP max_ that could be achieved by a *K. phaffii* strain overexpressing the β-alanine pathway is significantly higher for cells growing on glucose (> 0.9 g_3-HP_ g_Glucose_^−1^) than for methanol-grown cells (0.795 g_3-HP_ g_MetOH_^−1^), based on simulations performed with the *K. phaffi*’s iMT1026 v3.0 genome-scale metabolic model [42]. Since there are no fundamental differences between *K. phaffii*’s and *S. cerevisiae*’s respiratory glucose metabolisms, the maximum theoretical 3-HP yields on glucose are very similar between these yeasts [37]. Overall, we demonstrate the potential of *K. phaffii* to achieve 3-HP yields on methanol comparable to those obtained from glucose, albeit methanol assimilation being less energy efficient. Furthermore, the volumetric productivities calculated for the fed-batch phase in this study are more than twofold higher than the one obtained for the above-mentioned *S. cerevisiae* strain (0.21 g l^−1^ h^−1^, calculated from data given by Borodina et al. [37]).

Production of 3-HP from methanol has also been reported by an engineered *Methylobacterium extorquens* AM1 expressing the reductive malonyl-CoA pathway [48], although the yields obtained were an order of magnitude smaller than the ones reported in this study. Furthermore, the volumetric productivities achieved herein are remarkably higher (about 3.2-fold) to the productivities observed in the recently published *K. phaffii* strain producing 3-HP from sole methanol (0.15 g l^−1^ h^−1^, calculated from data reported by Wu et al. [10]). To our knowledge, the values obtained in this study are the highest volumetric productivities reported so far for a 3-HP production process based on the β-alanine pathway.

## Future perspectives

Although the 3-HP production yields and productivities we demonstrate herein are promising, further strain and process engineering is clearly needed to reach economically feasible metrics for carboxylic acid production at industrial scale, that is, a productivity and product yield not lower than 2.5 g l^−1^ h^−1^ and 0.5 g g^−1^, respectively [16]. Moreover, downstream operations may account for 20 to 60% of the entire production process costs. To reduce the overall separation and purification expenses, a final product concentration in the range of 50–100 g l^−1^ would be required for process scale up [49].

Despite the great potential of *K. phaffii* to produce metabolites such as 3-HP from methanol, a substantial fraction of the methanol consumed by the cells (typically, up to 50% or even higher) is channeled through the dissimilatory pathway to CO_2_, limiting the carbon flux entering the assimilation pathway to pyruvate [50], an important central metabolite as well as the critical precursor for 3-HP production. Thereby, metabolic engineering strategies aiming to shift the carbon flux split ratio between the assimilatory and dissimilatory pathways may push carbon flux into 3-HP production and reduce the carbon loss caused by the direct oxidation of methanol into CO_2_ in *K. phaffii*. In addition, pulling pyruvate flux into the β-alanine pathway, e.g., by enhancing the generation of pathway precursors such as oxaloacetate and aspartate, can also be effective strategy. Full exploitation of the improved supply of NADPH by overexpression of the NADP^+^-dependent formate dehydrogenase gene described in this study probably requires further fine-tuning of the *ydfG* gene expression levels for enhanced 3-HP production. Besides, final product concentration demands for industrial scale production will probably require engineering *K. phaffii*’s tolerance for high 3-HP concentrations, as demonstrated for other yeasts.

## Conclusions

In this study, we successfully engineered the synthetic β-alanine to 3-HP pathway in *K. phaffii* for the first time. Minimal pathway optimization led to a final 3-HP titer of 21.4 g l^−1^, a 0.15 g g^−1^ 3-HP yield on methanol, and a volumetric productivity of 0.48 g l^−1^ h^−1^ in controlled fed-batch cultures. Notably, no major by-products were observed in the fermentation broth. Overexpression of a mutated formate dehydrogenase from *Pseudomonas* sp. 101 aiming at increasing NADPH availability led to a higher carbon uptake rate in *K. phaffii*. Exometabolome profiling points at the pyruvate node as a potential target for future metabolic engineering. Although further strain and process optimization is required to make this system industrially attractive, we laid the basis for development of yeast strains for 3-HP production from renewable C1 feedstocks.

## Materials and methods

### Computational methods

The genome-scale metabolic model of *K. phaffii* used for all the simulations performed in this study was the iMT1026 v3.0 [42]. To evaluate the Υ_3−HP max_ of the malonyl-CoA and the β-alanine pathways, a set of experimental and calculated growth parameters based on data obtained from chemostat methanol cultivations were defined as constraints. When growing on this substrate, the growth associated and the non-growth associated maintenance energy (GAME and NGAME parameters) were calculated to be 166.77 mmol _ATP_ g_DCW_^−1^ and 0.44 mmol _ATP_ g_DCW_^−1^ h^−1^, respectively. The maximum specific substrate uptake rate (q_MetOH max_) was determined to be 7.82 mmol _MetOH_ g_DCW_^−1^ h^−1^ at µ_max_ = 0.10 h^−1^ [42]. The reactions for 3-HP production and excretion using either the malonyl-CoA or β-alanine pathways were added to the original model. The 3-HP yields (Υ_3-HP_) simulating different oxygen availability conditions (q_O2_) and growth rates (µ) were calculated using the COBRA Toolbox v3.0 [51], and the colored phase planes were generated using the ‘*pcolor*’ function from Matlab R2021a (Mathworks, Inc., Natick, MA, USA).

For the experimental data validation, the main physiological macroscopic parameters derived from the methanol fed-batch cultivations were used as constraints, together with the calculated NGAME value for methanol growth, and 3-HP production was maximized by performing a parsimonious enzyme usage Flux Balance Analysis (pFBA) using the COBRA Toolbox v3.0 software [51] in Matlab R2020b (Mathworks, Inc., Natick, MA, USA).

### Plasmid and strain construction

The parental strain *K. phaffii* CBS7435 (CBS, Centraalbureau voor Schimmelcultures, Utrecht, NL) was used to generate a battery of 3-HP-producer strains by means of CRISPR/Cas9-mediated homology-directed integrations of the heterologous genes. Plasmids and strains used in this study are listed in Table 3 from this section. The heterologous genes encoding for an aspartate-1-decarboxylase from *T. castaneum* (PAND_Tc_, *GenBank: ABU25221*), a β-alanine-pyruvate aminotransferase from *B. cereus* (BAPAT_Bc_, *GenBank: EEL86940*), a 3-hydroxypropionate dehydrogenase from *E. coli* (YDFG_Ec_, *GenBank: 12932746*), and a mutated formate dehydrogenase from *Pseudomonas* sp. (strain 101) (FDH(V9)_Pse(A199G/D222Q/S381V/C256A/H380K)_, *UniprotKB: P33160*), were codon-optimized and synthesized by GenScript Biotech (Piscataway, NJ, USA) for its expression in *K. phaffii* (*GenBank* accession numbers: OR360384, OR360385, OR360386, and OR360387, respectively).

**Table 3:** List of plasmids and strains used in this study

Plasmids/strains	Modules/genotype	References
Plasmids		
BB1_12_p*AOX1*	P_*AOX1*_, KanR^+^	[5]
BB1_12_p*FDH1*	P_*FDH1*_, KanR^+^	
BB1_12_p*POR1*	P_*POR1*_, KanR^+^	
BB1_12_p*PDC1*	P_*PDC1*_, KanR^+^	
BB1_23	Ø, KanR^+^	
BB1_34_*RPS3*tt	_*RPS3*_tt, KanR^+^	
BB1_34_Sc*CYC1*tt	_Sc*CYC1*_tt, KanR^+^	
BB1_34_*TDH3*tt	_*TDH3*_tt, KanR^+^	
BB2_BC	Ø, AmpR^+^	
pCC1-4k_TcPAND	*panD*^+^, *T. castaneum* gene encoding for PAND_Tc_ enzyme	GenScript Biotech
pCC1-4k_BcBAPAT	*yhxA*^+^, *B. cereus* gene encoding for BAPAT_Bc_	
BB1_23_EcYDFG	*ydfG*^+^, *E. coli* str. K-12 gene encoding for YDFG_Ec_	
BB1_23_PseFDH(V9)	*fdh*^+^, *Pseudomonas* sp. (strain 101) gene encoding for FDH_Pse_(V9)_(A199G/D222Q/S381V/C256A/H380K)_	
BB2_BC_TcPAND	P_*AOX1*_-PAND_Tc_-*RPS3*tt	This work
BB2_BC_BcBAPAT	P_*FDH1*_-BAPAT_Bc_-Sc*CYC1*tt	
BB2_BC_EcYDFG	P_*POR1*_-YDFG_Ec_-*TDH3*tt	
	P_*PDC1*_-YDFG_Ec_-*TDH3*tt	
BB2_BC_PseFDH(V9)	P_*FDH1*_-FDH(V9)_Pse_-*TDH3*tt	
BB3nK_ext_AD	Ø, KanR^+^	[6]
BB3nK_ext_AD_TcPAND	5′-HR_P_*AOX1*_-PAND_Tc_-*RPS3*tt_3′-HR(*RGI2*)	This work
	5′-HR_P_*AOX1*_-PAND_Tc_-*RPS3*tt_3′-HR(p*GAP*^UP^)	
BB3nK_ext_AD_BcBAPAT	5′-HR_P_*FDH1*_-BAPAT_Bc_-Sc*CYC1*tt_3′-HR(p*TEF1-α*^UP^)	
BB3nK_ext_AD_EcYDFG	5′-HR_P_*POR1*_-YDFG_Ec_-*TDH3*tt_3′-HR(p*FLD1*^UP^)	
	5′-HR_P_*PDC1*_-YDFG_Ec_-*TDH3*tt_3′-HR(p*FLD1*^UP^)	
BB3nK_ext_AD_PseFDH(V9)	5′-HR_P_*FDH1*_-FDH(V9)_Pse_-*TDH3*tt_3′-HR(p*AOX1*^UP^)	
BB3cN_p*GAP*_23*_pLAT1_Cas9	P_*GAP*__Ø_P_LAT1__Cas9, NrsR^+^	[6]
BB3cK_p*GAP*_23*_pLAT1_Cas9	P_*GAP*__Ø_P_LAT1__Cas9, KanR^+^	
BB3cH_p*GAP*_23*_pLAT1_Cas9	P_*GAP*__Ø_P_LAT1__Cas9, HygR^+^	
BB3cK_p*GAP*_gRNA1(*RGI2*)_pLAT1_Cas9	P_*GAP*__gRNA1(*RGI2*)_P_LAT1__Cas9, KanR^+^	This work
BB3cH_p*GAP*_gRNA1(p*GAP*^UP^)_pLAT1_Cas9	P_*GAP*__gRNA1(P_*GAP*_^UP^)_P_LAT1__Cas9, HygR^+^	
BB3cK_p*GAP*_gRNA2(p*TEF1-α*^UP^)_pLAT1_Cas9	P_*GAP*__gRNA2(P_*TEF1-α*_^UP^)_P_LAT1__Cas9, KanR^+^	
BB3cN_p*GAP*_gRNA1(p*FLD1*^UP^)_pLAT1_Cas9	P_*GAP*__gRNA1(P_*FLD1*_^UP^)_P_LAT1__Cas9, NrsR^+^	
BB3cN_p*GAP*_gRNA1(p*AOX1*^UP^)_pLAT1_Cas9	P_*GAP*__gRNA1(P_*AOX1*_^UP^)_P_LAT1__Cas9, NrsR^+^	
*K. phaffii* strains		
CBS7435	Wild-type	CBS, Centraalbureau voor Schimmelcultures
PpCβ10	P_*AOX*1__PAND_Tc_ + P_*FDH1*__BAPAT_Bc_ + P_*POR1*__YDFG_Ec_	This work
PpCβ20	P_*AOX1*__PAND_Tc_ + P_*FDH1*__BAPAT_Bc_ + P_*PDC1*__YDFG_Ec_	
PpCβ11	2x(P_*AOX1*__PAND_Tc_) + P_*FDH1*__BAPAT_Bc_ + P_*POR1*__YDFG_Ec_	
PpCβ21	2x(P_*AOX1*__PAND_Tc_) + P_*FDH1*__BAPAT_Bc_ + P_*PDC1*__YDFG_Ec_	
PpCβ20-P	PpCβ20 + P_*FDH1*__FDH(V9)_Pse_	
PpCβ21-P	PpCβ21 + P_*FDH1*__FDH(V9)_Pse_	

The modular cloning vectors used in this work are described in Prielhofer et al. [5], and are available at Addgene (Watertown, MA, USA) as Golden*Pi*CS kit. The CRISPR/Cas9-mediated homology-directed genome editing protocol performed in this study is described in Gassler et al. [6], and a ready-to-use plasmid kit named CRIS*Pi* can also be found at Addgene. Further description of the molecular cloning protocols followed in this study can be found in the Additional file 5.

Different genomic regions were selected as targets to efficiently design each sgRNA, focusing on intergenic regions such as upstream of the promoters, where the integration of exogenous genes usually leads to stable expression, and cell fitness remains unaffected [52]. Accordingly, the BAPAT_Bc_ expression cassette was targeted within the 100-bp upstream of *TEF1*-α promoter (PP7435_Chr1-1535), while the YDFG_Ec_ expression unit was located within the first 100-bp of P_*FLD1*_ (PP7435_Chr3-0140), hence confirming this target sequence as a non-critical region of the *FLD1* promoter. The PAND_Tc_ expression cassette was integrated into the *RGI2* locus [5], almost 800-bp upstream of a hypothetical protein (PP7435_Chr1-0725). The second copy of the PAND_Tc_ donor DNA template was targeted within 50-bp upstream of the *GAP* promoter (PP7435_Chr2-0858) for homology-directed DNA repair. Finally, the PseFDH(V9) expression cassette was targeted within 50-bp upstream of the *AOX1* promoter (PP7435_Chr4-0130). The potential sgRNA candidates binding target regions within the selected loci were assessed with CHOPCHOP, a widely used web tool for CRISPR-based genome editing [53]. CHOPCHOP v2 seeks and scores suitable 20 nucleotide target sites, called protospacers, followed by a 5′-NGG-3′ motif (PAM sequence). The existence of four consecutive thymine bases (TTTT) within the protospacer sequence might cause Pol III termination. Also, GC content without PAM sequence should be 20–80% [54]. Considering that the choice of the sgRNA causes locus-specific variations on targeting efficiency, and even some sgRNAs might not be functional, two high-scored sgRNA targets for each genome locus were selected for the experiments (Table 4), eventually using one to perform each CRISPR/Cas9-mediated gene insertion into the *K. phaffii* genome.

**Table 4:** Variable sgRNA targets custom-designed in this study

Target name	Locus	Sequence (5′→3′)	Expression cassette
p*TEF1-α*^UP^_sgRNA1	PP7435_Chr1-1535	AACAACACTAAACTACCTTG	P_*FDH1*__BAPAT_Bc__Sc*CYC1*tt
p*TEF1-α*^UP^_sgRNA2		**TTAAGGATGTGTAGTGTCAA**	
p*FLD1*^UP^_sgRNA1	PP7435_Chr3-0140	**TGCTAATGGTAGTTATCCAA**	P_*POR1*_-YDFG_Ec_-*TDH3*tt P_*PDC1*_-YDFG_Ec_-*TDH3*tt
p*FLD1*^UP^_sgRNA2		CTATAGGATAAAAACAGGAG	
*RGI2*_sgRNA1	PP7435_Chr1-0725	**TCTCAACGTATTTATATGGT**	P_*AOX1*_-PAND_Tc_-*RPS3*tt
*RGI2*_sgRNA2		ATGAAGCCACTTCAACTACG	
p*GAP*^UP^_sgRNA1	PP7435_Chr2-0858	ATCGATAATAGTCGCATGTG	P_*AOX1*_-PAND_Tc_-*RPS3*tt
p*GAP*^UP^_sgRNA2		**CGTTAGGTCAGTGATGACAA**	
p*AOX1*^UP^_sgRNA1	PP7435_Chr4-0130	**ATTGTGAAATAGACGCAGAT**	P_*FDH1*_-FDH(V9)_Pse_-*TDH3*tt
p*AOX1*^UP^_sgRNA2		GCAGTCGATCTCAAAAGCAA	

The sgRNA target used for each genomic insertion is highlighted in bold

Electrocompetent *K. phaffii* cells were prepared as described elsewhere [55]. Donor DNA plasmids carrying each expression cassette flanked by the corresponding homology arms were linearized using the FastDigest BpiI (IIs class) (Thermo Fisher Scientific, Waltham, MA, USA). Transformation was performed according to the CRISPR/Cas9-mediated homology-directed genome editing protocol for *K. phaffii* [6]. Correct genomic insertions were verified by Sanger sequencing using a set of primers covering the 5′ and the 3′ homology arms (see Additional file 5: Table S2). The sequenced clones were confirmed to be genetically identical. Recombinant yeast strains were grown at 30 °C in YPD agar plates (1% yeast extract, 2% peptone, 2% dextrose and 15 g l^−1^ agar). The medium was supplemented with the appropriate antibiotic when required: Nourseothricin (200 µg ml^−1^ working concentration for *K. phaffii*) from Dismed S.A. (Asturias, Spain), and Geneticin (500 µg ml^−1^) or Hygromycin (200 µg ml^−1^) from InvivoGen (San Diego, CA, USA).

### Screening in deep-well plates

*Komagataella phaffii* strains were grown overnight in 24 deep-well plates containing YPG medium (1% yeast extract, 2% peptone and 1% v/v glycerol). Cultures were placed on a platform with a slope of 20° in an incubator shaker Multitron Standard (Infors HT, Bottmingen, Switzerland) with a 2.5 cm orbit, and grown at 30 °C and 220 rpm. Afterwards, a 24 deep-well plate containing 2 ml of buffered minimal methanol medium (BMM; 100 mM potassium phosphate buffer pH 6, 1.34% yeast nitrogen base (YNB), 0.4 mg l^−1^ biotin and 0.5% v/v pure methanol) was inoculated with the overnight cultures, at a starting OD_600_ of 0.1. The cultures were grown for 48 h at 25 °C. Moreover, the relative humidity (rh) in the incubation chamber was fixed to 80%. Agitation was set to 220 rpm. After 24 h, 1% v/v pure methanol pulse was added to the cultures. Three independent transformants from each strain were tested to discard any biological variability. The parental strain *K. phaffii* CBS7435 was used as a negative control. All clones were inoculated in triplicate. At the end of the culture, the final biomass concentration of each deep-well was determined in duplicate with a 96-well microtiter plate using a Multiskan FC Microplate Photometer (Thermo Fisher Scientific, Waltham, MA, USA) to ensure all the cultures were grown up to a similar endpoint OD_600_.

### Shake-flask cultures for small-scale growth kinetics experiments

*Komagataella phaffii* strains were inoculated from cryo-vials into 5 ml of YPG medium in 50 ml Falcon tubes. Cultures were grown overnight at 30 °C and 180 rpm. The overnight cultures were then diluted to an OD_600_ of 0.2 in 250 ml shake flasks containing 25 ml of BMM and grown in an incubator shaker Multitron Standard for 12 h at 25 °C, 180 rpm and a relative humidity of 80%, to adapt cells to methanol metabolism and setting the screening conditions. Afterwards, these cultures were used to inoculate 250 ml shake flasks containing 25 ml of fresh BMM at a starting OD_600_ of 0.2 and grown during 24 h at the same cultivation conditions. Samples were taken every 3 h to measure the OD_600_ in duplicate with a 96-well microtiter plate using the SPECTROstar Nano absorbance microplate reader (BMG Labtech, Ortenberg, Germany).

### Bioreactor cultivations

Fed-batch cultures were performed in duplicate using a DASGIP Parallel Bioreactor System (Eppendorf, Germany). The starting volume of each 1.3 l reactor vessel was 500 ml. The batch medium consisted of 40 g l^−1^ glycerol, 1.8 g l^−1^ citric acid, 0.02 g l^−1^ CaCl_2_·2H_2_O, 12.6 g l^−1^ (NH_4_)_2_HPO_4_, 0.5 g l^−1^ MgSO_4_·7H_2_O, 0.9 g l^−1^ KCl, 50 µl antifoam Glanapon 2000 kz (Bussetti and Co GmbH, Vienna, Austria), 0.4 mg l^−1^ biotin, 2 ml l^−1^ of vitamin stock solution [47], and 5 ml l^−1^ of PTM1 trace salts [45]. The pH was adjusted to 5 using 5 M HCl. The vitamins, the biotin and the trace salts were filter-sterilized and added to the autoclaved medium after cooling down.

Inocula were prepared as described elsewhere [56]. Reactors were inoculated at a starting OD_600_ of 1. The pH was controlled at 5 throughout the culture using 15% ammonia (only base addition was used). The temperature was set to 28 °C, and the inlet gas was fed into the reactors at an aeration rate of 1 vvm (0.5 l min^−1^). The dissolved oxygen (DO) was set to 30%, automatically controlled using the following cascade: (1) Increasing the stirring rate from 400 to 1000 rpm; (2) Raising the percentage of oxygen in the inlet gas, while maintaining an aeration rate of 0.5 l min^−1^. For the fed-batch phase, the temperature was set to 25 °C. Pure methanol (ρ = 792 g l^−1^) and feeding salts were added separately to avoid precipitation. The feeding salts medium composition was 0.35 g l^−1^ CaCl_2_·2H_2_O, 10 g l^−1^ KCl, 6.45 g l^−1^ MgSO_4_·7H_2_O, 200 µl antifoam Glanapon 2000 kz, 1.2 mg l^−1^ biotin, 6 ml l^−1^ of vitamin stock solution, and 15 ml l^−1^ of PTM1 trace salts. This media was prepared at 2× concentration since pure methanol was used. Two separated solutions were prepared and autoclaved, one containing CaCl_2_·2H_2_O, and the other containing the rest of the components. After that, both solutions were mixed under sterile conditions at room temperature. The vitamins, the biotin and the trace salts were filter-sterilized and added to this final feeding solution.

After reaching a peak in the % of CO_2_ from the exhaust gas, indicating the end of the batch phase, two pulses of pure methanol (1 and 2 g l^−1^, respectively) were sequentially added into the reactors. Once the methanol of the last pulse was completely depleted, the cultures were fed with a constant feeding rate (F_o_) for 6 h to complete the transition phase. After that, the feeding medium was added to the bioreactors using a pre-programmed exponential feeding strategy for controlled specific growth rate described in the following equation:

$$F\left(t\right)= \frac{{\upmu }\left[X\left({t}_{o}\right)V\left({t}_{o}\right)\right]}{{Y}_{X/S}{S}_{o}}{e}^{\left[{\upmu }\left(t-{{t}_{o}}\right)\right]}$$

where µ was 0.03 h^−1^, the X_0_ was set to 22 g l^−1^ and the Y_X/S_ to 0.273 g g^−1^.

The reactors were sampled every 5.5 h to measure OD_600_, biomass dry cell weight and supernatant metabolites. The OD_600_ measurements were performed in triplicate using a Lange DR 3900 spectrophotometer (Hach, Loveland, CO, USA). The Relative Standard Deviation (RSD) was below 5%. For the biomass DCW determination, 10 ml of distilled water with 9 g l^−1^ NaCl were used to wet the pre-weighted glass microfiber filters (APFF04700, Merck Millipore) before filtering 2 ml of culture for each triplicate. After that, the filters were washed using the same volume of the NaCl solution and dried for 24 h at 105 °C. Filters containing the dry biomass were weighted to calculate the DCW. This parameter was quantified for three samples throughout the fed-batch, in which the RSD was below 2.5%. For the rest of the samples, the DCW was calculated by interpolation using an equation from a linear regression between DCW and OD_600_ measures of the initial fed-batch cultivations. To quantify the metabolites, 2 ml of culture samples were centrifuged 5 min at 13,400 rpm using a MiniSpin (Eppendorf, Germany). The supernatant was then filtered with a 0.2 μm pore size single-use syringe filter (SLLGX13NK, Merck Millipore, CA, USA). The filtered supernatant was stored at − 20 °C until HPLC analysis for methanol and 3-HP quantification, and for exometabolome profiling by NMR.

### Analytical methods

Methanol and 3-HP were quantified using an HPLC Dionex Ultimate3000 (Dionex – Thermo Fischer Scientific). The compounds were separated with an ionic exchange column ICSep ICE-COREGEL 87H3 (Transgenomic, Omaha, NE, USA) using 6 mM sulphuric acid as mobile phase at a flow rate of 0.6 ml min^−1^. Both metabolites were quantified from the Refractive Index (RI) spectrum.

For the NMR method, a Bruker Avance 600 MHz NMR spectrometer operating at a proton (^1^H) frequency of 600.13 MHz equipped with a triple-resonance Bruker TXI 5 mm room-temperature probe and an autosampler (Bruker Biospin, Rheinstetten, Germany) was utilized. The probe temperature was maintained at 298.0 K in all experiments. Once centrifuged and filtered, each supernatant aliquot (400 µl) was mixed with a D_2_O sodium phosphate buffer (200 µl, 0.2 M, pH 7.4) containing an internal standard (3-(trimethylsilyl)-[2,2,3,3-^2^H_4_]-propionic acid sodium salt (TSP), 1 mM) and transferred to the NMR tube. All samples were analysed conducting 1D ^1^H NMR experiments with presaturation of the residual water signal applying the pulse sequence commonly termed 1D NOESY-presat [57]. Data were collected into 32K data points during an acquisition time of 2.3 s using a recycle delay of 2 s. Spectra were recorded in the time domain as interferograms (FID) across a spectral width of 7211 Hz and as the sum of 1024 transients. Each FID was multiplied by an exponential apodisation function equivalent to a 0.2 Hz line broadening, prior to Fourier transform. The frequency-domain spectra were manually phased, baseline corrected, and referenced to the TSP resonance at δ_H_ 0.00 ppm. The identification of metabolites was carried out using the BMRB spectral database [58] and the software Chenomix NMR Suite 8.5 (Chenomix Inc., Edmonton, Canada).

### Process parameters determination and data consistency checking

Recorded offline and online state variables during the fed-batch phase of the bioreactor-scale experiments, namely biomass X, substrate S, product P, flow rate F, and initial volume V_0_ (see Additional files 4 and 6) were used to calculate all derived variables. Details of all equations derived from the mass balances used to calculate yields and rates in the fed-batch experiments can be found elsewhere [59, 60]. In short, intermediate values of the offline variables (X, P), corresponding to the time when online measured variables were taken, were calculated (interpolated) after application of the ‘*smoothingspline’* function from Matlab R2021a (Mathworks, Inc., Natick, MA, USA). Variables measured as concentrations in the liquid phase were referred to the total volume. Recorded state variables were used to calculate the accumulated variables (total amounts, XV, SV, PV, V) during the fed-batch phase. Linear regression was applied on the time evolution of accumulated variables. Regression slopes and its confidence intervals were taken as averaged rates of total state variables. Averaged rates of state variables during the fed-batch phase were used to calculate the derived variables (growth rate µ, q-rates, and yields). The carbon and electron balances were verified using the averaged measures prior to apply common reconciliation techniques [61]. For the elemental mass balance verification, the mean elemental biomass composition CH_1.88_O_0.63_N_0.20_S_0.004_ with an ash content of 5.9%, previously reported for cells growing on methanol at µ = 0.035 h^−1^ [42], was assumed in this study. Loss of methanol by stripping was considered almost negligible since C-balances were satisfied with less than 6% of deviation. Statistical χ2 consistency test based on h-index, applied on measured data balances as described elsewhere [62, 63], was passed with a confidence level of 95%. Consequently, there was no evidence for gross measurement errors. The method used for this purpose is also described in detail elsewhere [59].

### Supplementary Information

**Additional file 1.** Diagrams and stoichiometric balances of the malonyl-CoA and β-alanine pathways. **Figure S1.** Glucose, glycerol, and methanol metabolization to 3-HP via the two main metabolic pathways reported in yeast. **Table S1.** Stoichiometric analysis of the main metabolic pathways towards 3-HP using different carbon sources.

**Additional file 2.** Raw data from the 24-deep well plate cultivations and cell growth kinetics experiments.

**Additional file 3.** NMR spectra for PpCβ20, PpCβ21 and PpCβ21-P strains exometabolome. **Figure S2.** ^1^H NMR spectra of PpCβ20 strain supernatant samples at different times of the methanol-feeding phase. **Figure S3.**^1^H NMR spectra of PpCβ21 strain supernatant samples at different times of the methanol-feeding phase. **Figure S4.** ^1^H NMR spectra of PpCβ21-P strain supernatant samples at different times of the methanol-feeding phase.

**Additional file 4.** Raw and processed data obtained from the bioreactor-scale experiments.

**Additional file 5.** Molecular cloning materials and methods. **Table S2.** List of primers used for amplification and cloning of DNA parts, sgRNAs construction, and sequence verification of integrations.

**Additional file 6.** Raw data of the online monitored standard process parameters.

## Data Availability

The data that supports the findings of this study are included in this published article/Additional files. Further datasets used and/or analyzed during the current study are available from the corresponding author on reasonable request.
