# Supplementary material for: Engineering the synthetic β-alanine pathway in Komagataella phaffii for conversion of methanol into 3-hydroxypropionic acid
Source: Microb Cell Fact. 2023 Nov 17;22:237. doi: 10.1186/s12934-023-02241-9 (PMC10655335; doi:10.1186/s12934-023-02241-9)
Supplement: Supplementary file 1 — Additional file 1. Diagrams and stoichiometric balances of the malonyl-CoA and β-alanine pathways. Figure S1. Glucose, glycerol, and methanol metabolization to 3-HP via the two main metabolic pathways reported in yeast. Table S1. Stoichiometric analysis of the main metabolic pathways towards 3-HP using different carbon sources. [file 12934_2023_2241_MOESM1_ESM.docx]

**Glucose**

C_6_H_12_O_6_

**Glycerol**

C_3_H_8_O_3_

**Methanol**

CH_3_OH

Glyceraldehyde-3-phospate (G3P)

C_3_H_7_O_6_P

Pyruvate

C_3_H_4_O_3_

Oxaloacetate

C_4_H_4_O_5_

Acetaldehyde

C_2_H_4_O

L-aspartate

C_4_H_7_NO_4_

β-alanine

C_3_H_7_NO_2_


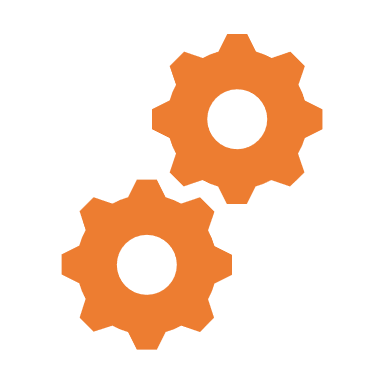


Malonate semialdehyde

C_3_H_4_O_3_

3-hydroxypropionic acid

C_3_H_6_O_3_


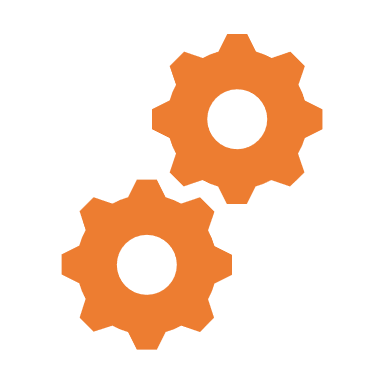

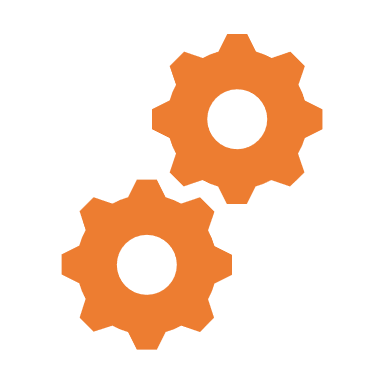


Acetyl-CoA

C_23_H_38_N_7_O_17_P_3_S

Malonyl-CoA

C_24_H_38_N_7_O_19_P_3_S

Acetate

C_2_H_4_O_2_


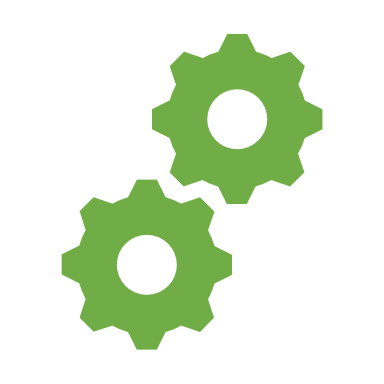


**Fig. S1** Glucose, glycerol and methanol metabolization to 3-HP via the two main metabolic pathways reported in yeast. The black arrows indicate the native reactions of *K. phaffii* metabolism. In the malonyl-CoA pathway (left), the green arrow indicates the malonyl-CoA conversion to 3-HP through two sequential reactions catalyzed by a heterologous bi-functional malonyl-CoA reductase (MCR_Ca_) from *C. aurantiacus*. In the β-alanine pathway (right), the orange arrows indicate the three heterologous reactions from L-aspartate to 3-HP performed by an aspartate-1-decarboxylase (PAND_Tc_) from *T. castaneum*, a β-alanine-pyruvate aminotransferase (BAPAT_Bc_) from *B. cereus*, and a 3-hydroxypropionate dehydrogenase (YDFG_Ec_) from *E. coli*.

| ATP Synthase: NADH + H^+^ 🡪 NAD^+^ + 2.5 ATP | | |
| --- | --- | --- |
| Redox balancing mechanisms: NADPH 🡪 NADH | | |
| **GLUCOSE** | Malonyl-CoA pathway | 0.5 Glucose + 2 ATP 🡪 3-HP |
|  | β-alanine pathway | 0.5 Glucose 🡪 3-HP |
| **METHANOL** | Malonyl-CoA pathway | 3 Methanol + 4 ATP 🡪 3-HP |
|  | β-alanine pathway | 3 Methanol + 2 ATP 🡪 3-HP |
| **GLYCEROL** | Malonyl-CoA pathway | Glycerol 🡪 3-HP + 0.5 ATP |
|  | β-alanine pathway | Glycerol 🡪 3-HP + 2.5 ATP |

**Table S1** Stoichiometric analysis of the main metabolic pathways towards 3-HP using different carbon sources.

Adapted from [1]

1. Fina A, Brêda GC, Pérez-Trujillo M, Freire DMG, Almeida RV, Albiol J, et al. Benchmarking recombinant Pichia pastoris for 3-hydroxypropionic acid production from glycerol. Microb Biotechnol. 2021;14:1671–82.
