# Supplementary material for: Engineering the synthetic β-alanine pathway in Komagataella phaffii for conversion of methanol into 3-hydroxypropionic acid
Source: Microb Cell Fact. 2023 Nov 17;22:237. doi: 10.1186/s12934-023-02241-9 (PMC10655335; doi:10.1186/s12934-023-02241-9)
Supplement: Supplementary file 3 — Additional file 3. NMR spectra for PpCβ20, PpCβ21 and PpCβ21-P strains exometabolome. Figure S2. 1H NMR spectra of PpCβ20 strain supernatant samples at different times of the methanol-feeding phase. Figure S3.1H NMR spectra of PpCβ21 strain supernatant samples at different times of the methanol-feeding phase. Figure S4. 1H NMR spectra of PpCβ21-P strain supernatant samples at different times of the methanol-feeding phase. [file 12934_2023_2241_MOESM3_ESM.docx]

**Fig. S2** ^1^H NMR spectra of PpCβ20 strain supernatant samples at different times of the methanol-feeding phase. **a** early, **b** mid and **c** late. Assignment of signals corresponding to 3HP, methanol (1), 3-methyl-2-oxopentanoate (2), 2-oxoisocaproate (3) and ethanol (4) are indicated. Experiments acquired at a magnetic field of 600 MHz and at 298.0 K of temperature.


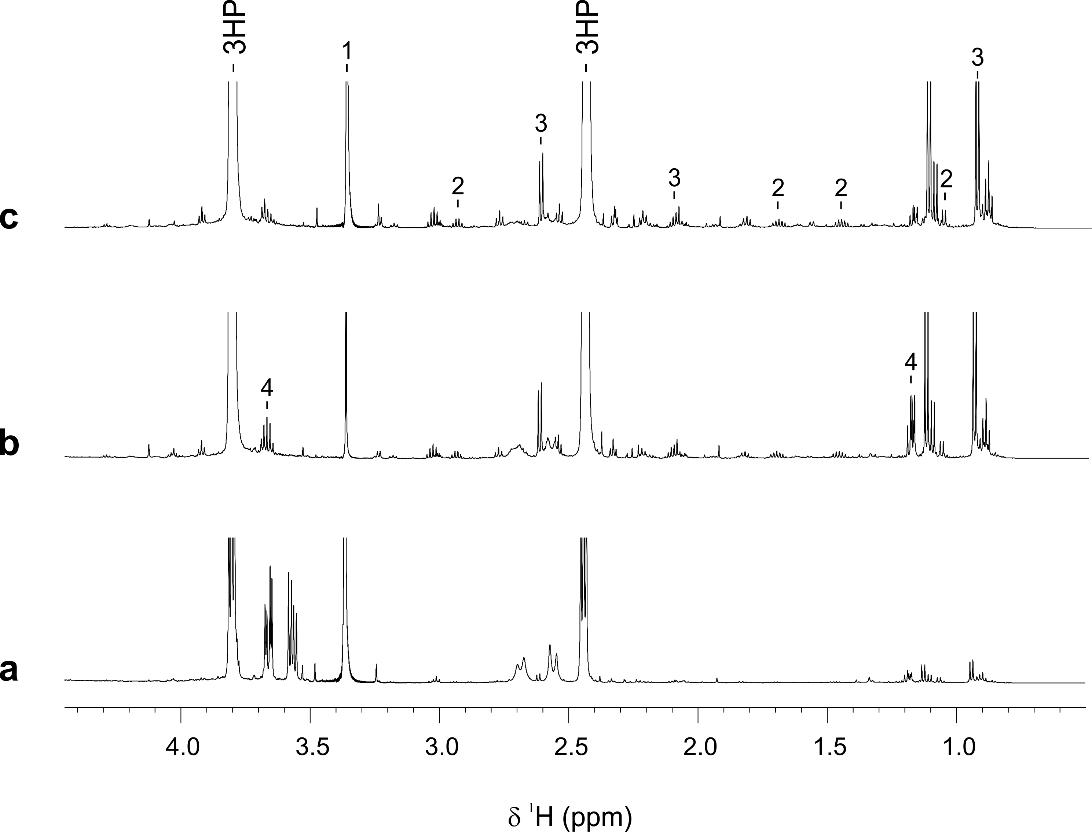

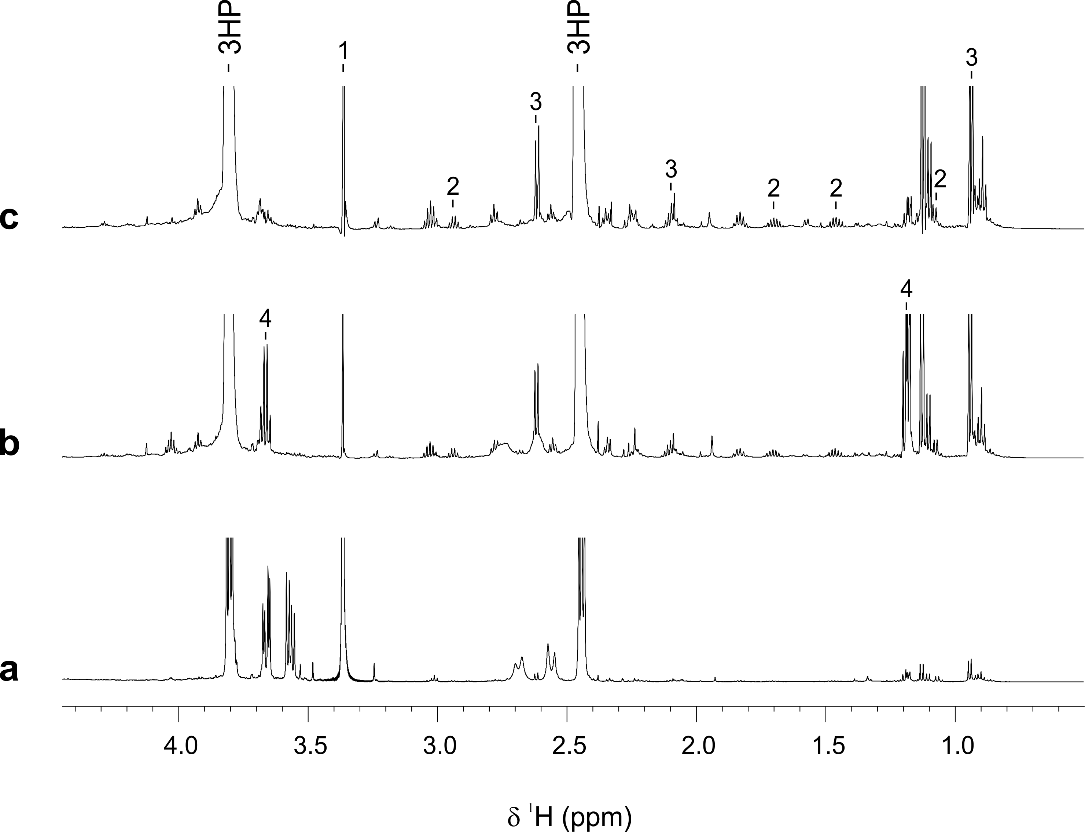


**Fig. S3** ^1^H NMR spectra of PpCβ21 strain supernatant samples at different times of the methanol-feeding phase. **a** early, **b** mid and **c** late. Assignment of signals corresponding to 3HP, methanol (1), 3-methyl-2-oxopentanoate (2), 2-oxoisocaproate (3) and ethanol (4) are indicated. Experiments acquired at a magnetic field of 600 MHz and at 298.0 K of temperature.


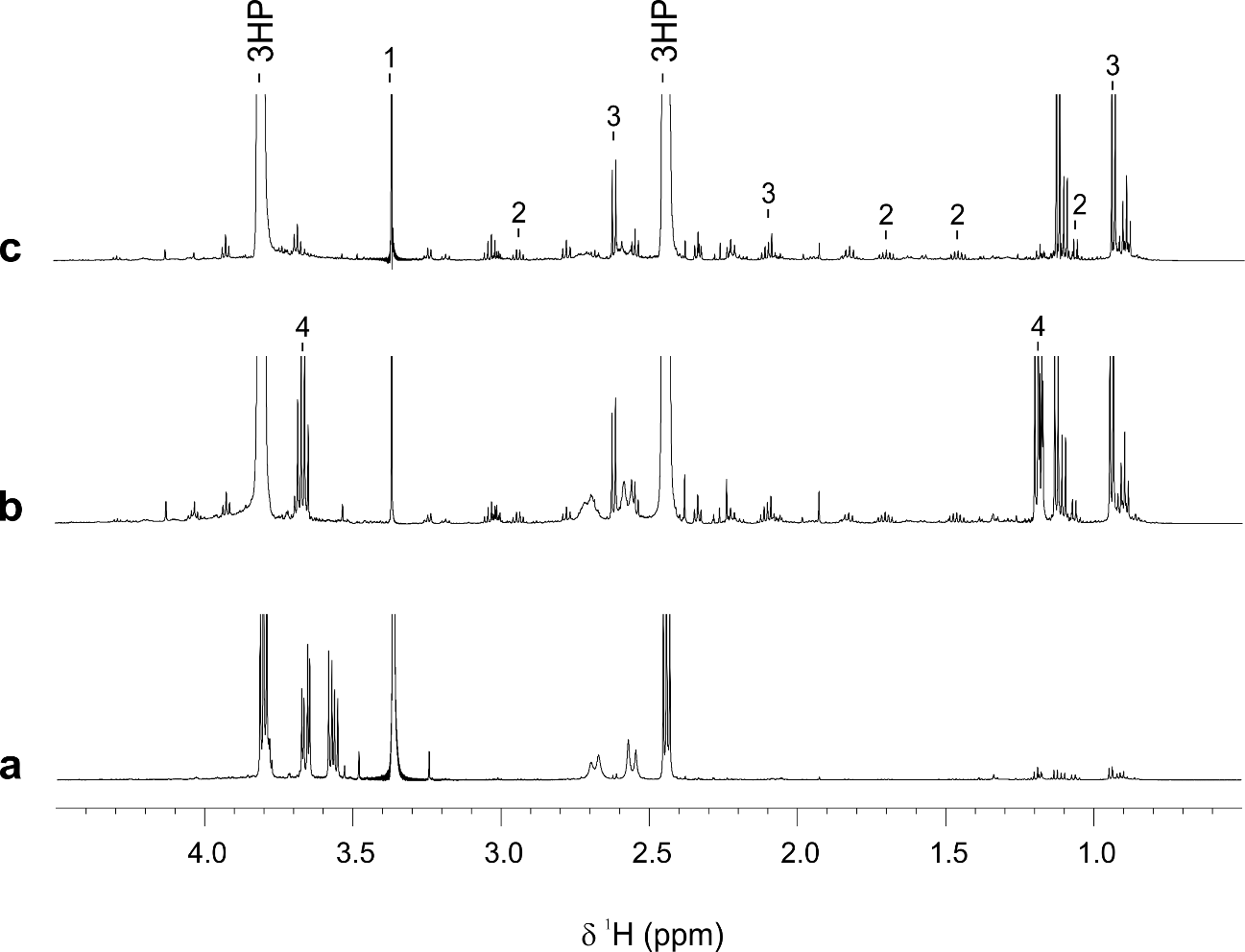


**Fig. S4** ^1^H NMR spectra of PpCβ21-P strain supernatant samples at different times of the methanol-feeding phase. **a** early, **b** mid and **c** late. Assignment of signals corresponding to 3HP, methanol (1), 3-methyl-2-oxopentanoate (2), 2-oxoisocaproate (3) and ethanol (4) are indicated. Experiments acquired at a magnetic field of 600 MHz and at 298.0 K of temperature.
