# Supplementary material for: Engineering the synthetic β-alanine pathway in Komagataella phaffii for conversion of methanol into 3-hydroxypropionic acid
Source: Microb Cell Fact. 2023 Nov 17;22:237. doi: 10.1186/s12934-023-02241-9 (PMC10655335; doi:10.1186/s12934-023-02241-9)
Supplement: Supplementary file 5 — Additional file 5. Molecular cloning materials and methods. Table S2. List of primers used for amplification and cloning of DNA parts, sgRNAs construction, and sequence verification of integrations. [file 12934_2023_2241_MOESM5_ESM.docx]

The *ydfG* gene encoding for a 3-hydroxypropionate dehydrogenase from *E. coli* K-12, and the *fdh* gene encoding for a mutated formate dehydrogenase from *Pseudomonas* sp. 101 were synthesized, codon optimized for its expression in *K. phaffii*, and cloned into the backbone BB1_23 from the Golden*Pi*CS kit (Addgene, MA, USA) by GenScript (NJ, USA) to obtain the plasmids BB1_23_EcYDFG and BB1_23_PseFDH(V9), respectively. The same procedure was followed for the *panD* and *yhxA* genes, encoding for an aspartate-1-decarboxylase from *T. castaneum* and a β-alanine-pyruvate aminotransferase from *B. cereus*, respectively, both cloned into the backbone pCC1-4k on this occasion, obtaining the plasmids pCC1-4k_TcPAND and pCC1-4k_BcBAPAT, respectively. All genes were flanked by *BbsI* restriction sites for further subcloning procedures using the Golden*Pi*CS kit and protocols [1], allowing the assembly of promoters, genes, and terminators in recipient BB2 by performing a Golden Gate Assembly (GGA) reaction using the enzyme *Bbs*I-HF® from New England Biolabs (NEB) (MA, USA). If necessary, the coding sequences of the genes were modified to remove all the internal *Bbs*I and *Bsa*I restriction sites, while maintaining a high codon usage.

A list of all the primers used in this study is available in Table S2 from this additional file. All the primers were ordered from Integrated DNA Technologies (IA, USA).

For gene insertions by homology-directed repair mediated by CRISPR/Cas9, a donor DNA template containing homologous regions (HR) must be provided. Following the CRISPR/Cas9-mediated genome editing protocol for replacements in *K. phaffii* [2], genomic regions consisting of 1000 bp upstream (5’-HR) and downstream (3’-HR) of the 20 bp target site were amplified by PCR using the Q5® High-Fidelity 2X Master Mix (NEB) following the protocol recommended by the polymerase provider, to finally fuse them with the corresponding heterologous expression cassette in between. If necessary, the HR sequences were modified to eliminate the internal *Bbs*I and *Bsa*I restriction sites to allow its subcloning. To do so, the following overlap extension PCR (or OE-PCR) protocol was used. First, two PCR reactions were performed using genomic DNA from *K. phaffii* CBS7435 strain as template: i) using the primers pair 3'-HR_*FLD1*^UP^_FW and 3'-HR_*FLD1*^UP^(Δ*Bbs*I)_RV, and ii) 3'-HR_*FLD1*^UP^(Δ*Bbs*I)_FW and 3'-HR_*FLD1*^UP^_RV (Table S2). Afterwards, the PCR products were purified from a 1% agarose gel, and mixed together in a new PCR reaction, without primers, using 100 ng of the longest fragment. The shortest fragment was added to the reaction using a molar ratio of 1:1. The required amount (ng) was calculated using the NEBioCalculator® (NEB). The annealing temperature of the homologous regions between the fragments was estimated with the NEB Tm Calculator (NEB). The overlap PCR was run for 15 cycles. For the extension step, the two primers flanking the outer parts, 3'-HR_*FLD1*^UP^_FW and 3'-HR_*FLD1*^UP^_RV, were added right after. The PCR was started again for 15 more cycles, using an annealing temperature matching the corresponding primers. Afterwards, the overlap extension product was gel-extracted and purified.

The four base pair overhangs added at the outer parts of the homologous regions allowed its assembly with the corresponding expression cassette in between in the recipient vector BB3nK_ext_AD, provided by the CRISP*i* kit from Addgene, by performing a GGA reaction using the enzyme *Bsa*I-HF®v2 from NEB. The external *Bbs*I sites also present in this BB3 plasmid enabled the excision of the final donor DNA construct by a simple overnight digestion using the *Bpi*I (*Bbs*I) restriction enzyme from Thermo Fisher Scientific (MA, USA).

Two sgRNAs were individually designed and generated for each target site and inserted into BB3 plasmids from the CRISP*i* kit following the protocols described in Gassler et al. [2].

The co-transformation of the donor DNA template and the BB3 plasmid harboring the sgRNA and a human codon optimized Cas9 for episomal expression in *K. phaffii* was performed as described elsewhere [2].

All the plasmids whose generation involved a PCR step were sequenced. Once the transformants were obtained, three isolates from each strain were checked individually at both 5’ and 3’ ends of the integrated donor DNA by Sanger sequencing using the verification primers (Table S2). The sequencing was performed by the Genomics and Bioinformatics Service of the Universitat Autònoma de Barcelona (Bellaterra, Spain).

**Table S2** List of primers used for amplification and cloning of DNA parts, sgRNAs construction, and sequence verification of integrations.

| Amplification of homology regions (HR) | | |
| --- | --- | --- |
| **Locus** | **Primer name** | **Primer sequence** |
| PP7435_Chr1-1535 | 5'-HR_p*TEF1-α*^UP^_FW | GATGGTCTCAGATCCTGAATGCGTGCTTAATAG |
|  | 5'-HR_p*TEF1-α*^UP^_RV | GATGGTCTCGCCGGTAACCAGATGCTGAGATTAAG |
|  | 3'-HR_p*TEF1-α*^UP^_FW | GATGGTCTCTAATTCACCTTTTAACCATCTTGCC |
|  | 3'-HR_p*TEF1-α*^UP^_RV | GATGGTCTCAAGCTATTTGACGGAGTCCATCTTG |
| PP7435_Chr3-0140 | 5'-HR_p*FLD1*^UP^_FW | GATGGTCTCAGATCGTCTTACCAGACAGACAG |
|  | 5'-HR_p*FLD1*^UP^_RV | GATGGTCTCACCGGGGGTGAACCTTGCTTATG |
|  | 3'-HR_p*FLD1*^UP^_FW | GATGGTCTCGAATTATTAGCACCGTGCCAGAG |
|  | 3'-HR_p*FLD1*^UP^(Δ*Bbs*I)_RV | CATAGTGACTATTGGATGACACTGCTATCA |
|  | 3'-HR_p*FLD1*^UP^(Δ*Bbs*I)_FW | GCAGTGTCATCCAATAGTCACTATGTT |
|  | 3'-HR_p*FLD1*^UP^_RV | GATGGTCTCGAGCTGTTCCCTCCATTCCAACAAT |
| PP7435_Chr1-0725 | 5'-HR_*RGI2*_FW | TTTTCGCGGTCTCCGATCGAGGTTTACAAGCTGTGATGTTCC |
|  | 5'-HR_*RGI2*_RV | TCCGGTGGTCTCCCCGGTGATGAACTGCCCGTCAAATTG |
|  | 3'-HR_*RGI2*_FW | TTTTCGCGGTCTCAAATTGAAGTGGCTTCATAATTTCAGAACTC |
|  | 3'-HR_*RGI2*_RV | TCCGGTGGTCTCCAGCTCGTTCGCTATATTATCATAGCCCAG |
| PP7435_Chr2-  0858 | 5'-HR_p*GAP*^UP^_FW | GATGGTCTCAGATCAAACGGTGATTTCGTTACCG |
|  | 5'-HR_p*GAP*^UP^_RV | GATGGTCTCGCCGGTGAAATCTTAAAATTGCCCC |
|  | 3'-HR_p*GAP*^UP^_FW | GATGGTCTCAAATTGGAACGGATGTCAAATGTCC |
|  | 3'-HR_p*GAP*^UP^_RV | GATGGTCTCGAGCTTGATCAGATCACATTCTTTCAC |
| PP7435_Chr4-0130 | 5'-HR_p*AOX1*^UP^_FW | GATGGTCTCAGATCCTGCTTAACAGGTTTAGGAATCTCGG |
|  | 5'-HR_p*AOX1*^UP^_RV | GATGGTCTCACCGGTCACAATACCAACATGAGTCAACC |
|  | 3'-HR_p*AOX1*^UP^_FW | CATGGTCTCAAATTCGGGAACACTGAAAAATAACAGTTATTATTCG |
|  | 3'-HR_p*AOX1*^UP^_RV | GATGGTCTCAAGCTATCCACCACCTAGAACTAGG |
| **sgRNAs construction** | | |
| **Locus** | **Primer name** | **Primer sequence** |
| PP7435_Chr1-1535 | 1_p*TEF1-α*^UP^_sgRNA1_FW | TGAAGACGCCATGGTTGTTCTGATGAGTCCGTGAGGACGAAACGAGTAAGCTCGTCAACA |
|  | 2_ p*TEF1-α*^UP^ _sgRNA1_FW | AAACGAGTAAGCTCGTCAACAACACTAAACTACCTTGGTTTTAGAGCTAGAAATAGCAAG |
|  | 1_p*TEF1-α*^UP^_sgRNA2_FW | TGAAGACGCCATGCCTTAACTGATGAGTCCGTGAGGACGAAACGAGTAAGCTCGTCTTAA |
|  | 2_p*TEF1-α*^UP^_sgRNA2_FW | AAACGAGTAAGCTCGTCTTAAGGATGTGTAGTGTCAAGTTTTAGAGCTAGAAATAGCAAG |
| PP7435_Chr3-0140 | 1_p*FLD1*^UP^_sgRNA1_FW | TGAAGACGCCATGTTAGCACTGATGAGTCCGTGAGGACGAAACGAGTAAGCTCGTCTGCT |
|  | 2_p*FLD1*^UP^_sgRNA1_FW | AAACGAGTAAGCTCGTCTGCTAATGGTAGTTATCCAAGTTTTAGAGCTAGAAATAGCAAG |
|  | 1_p*FLD1*^UP^_sgRNA2_FW | TGAAGACGCCATGCTATAGCTGATGAGTCCGTGAGGACGAAACGAGTAAGCTCGTCCTAT |
|  | 2_p*FLD1*^UP^_sgRNA2_FW | AAACGAGTAAGCTCGTCCTATAGGATAAAAACAGGAGGTTTTAGAGCTAGAAATAGCAAG |
| PP7435_Chr1-0725 | 1_*RGI2*_sgRNA1_FW | TGAAGACGCCATGTTGAGACTGATGAGTCCGTGAGGACGAAACGAGTAAGCTCGTCTCTC |
|  | 2_*RGI2*_sgRNA1_FW | AAACGAGTAAGCTCGTCTCTCAACGTATTTATATGGTGTTTTAGAGCTAGAAATAGCAAG |
|  | 1_*RGI2*_sgRNA2_FW | TGAAGACGCCATGCTTCATCTGATGAGTCCGTGAGGACGAAACGAGTAAGCTCGTCATGA |
|  | 2_*RGI2*_sgRNA2_FW | AAACGAGTAAGCTCGTCATGAAGCCACTTCAACTACGGTTTTAGAGCTAGAAATAGCAAG |
| PP7435_Chr2-0858 | 1_p*GAP*^UP^_sgRNA1_FW | TGAAGACGCCATGATCGATCTGATGAGTCCGTGAGGACGAAACGAGTAAGCTCGTCATCG |
|  | 2_p*GAP*^UP^_sgRNA1_FW | AAACGAGTAAGCTCGTCATCGATAATAGTCGCATGTGGTTTTAGAGCTAGAAATAGCAAG |
|  | 1_p*GAP*^UP^_sgRNA2_FW | TGAAGACGCCATGCTAACGCTGATGAGTCCGTGAGGACGAAACGAGTAAGCTCGTCCGTT |
|  | 2_p*GAP*^UP^_sgRNA2_FW | AAACGAGTAAGCTCGTCCGTTAGGTCAGTGATGACAAGTTTTAGAGCTAGAAATAGCAAG |
| PP7435_Chr4-0130 | 1_p*AOX1*^UP^_sgRNA1_FW | TGAAGACGCCATGCACAATCTGATGAGTCCGTGAGGACGAAACGAGTAAGCTCGTCATTG |
|  | 2_ p*AOX1*^UP^ _sgRNA1_FW | AAACGAGTAAGCTCGTCATTGTGAAATAGACGCAGATGTTTTAGAGCTAGAAATAGCAAG |
|  | 1_ p*AOX1*^UP^ _sgRNA2_FW | TGAAGACGCCATGGACTGCCTGATGAGTCCGTGAGGACGAAACGAGTAAGCTCGTCGCAG |
|  | 2_ p*AOX1*^UP^ _sgRNA2_FW | AAACGAGTAAGCTCGTCGCAGTCGATCTCAAAAGCAAGTTTTAGAGCTAGAAATAGCAAG |
| **Genome amplifications and sequence verification** | | |
| **Locus** | **Primer name** | **Primer sequence** |
| PP7435_Chr1-1535 | 5'-HR_p*TEF1-α*^UP^_out_FW | TTGGGAAGATGGCTGTACGG |
|  | 5'-HR_p*TEF1-α*^UP^_seq2 | CGAGGTGAACATTTCATAGG |
|  | p*FDH1*_BcBAPAT_in_RV | CGAAACGGCACGCTAAAGTTTCC |
|  | 3'-HR_p*TEF1-α*^UP^_out_FW | TACAGACGCGTGTACGCATG |
|  | 3'-HR_p*TEF1-α*^UP^_seq2 | ATTGGACACGTCGATGCTGG |
|  | 3'-HR_p*TEF1-α*^UP^_out_RV | ATCCCTTGTACCAGTCACAG |
| PP7435_Chr3-0140 | 5'-HR_p*FLD1*^UP^_out_FW | AGATGTCAGCCACCACAGTG |
|  | 5'-HR_p*FLD1*^UP^_seq2 | GACCTTCGGTAGACATTGTG |
|  | p*POR1*_EcYDFG_in_RV | GCAAAAATGGAATACGGTTGAGG |
|  | p*PDC1*_EcYDFG_in_RV | GATCTATTCTGTTGATGTTGATCCG |
|  | 3'-HR_p*FLD1*^UP^_out_FW | AGGCATTTGAGCATTGGC |
|  | 3’-HR_p*FLD1*^UP^_seq2 | GAGATATACGATTTGCGGC |
|  | 3'-HR_p*FLD1*^UP^_out_RV | GAGCCCAATCTGTTGTCC |
| PP7435_Chr1-0725 | 5'-HR_*RGI2*_out_FW | CGGCAACTGTTATCAGAATGC |
|  | 5’-HR_*RGI2*_seq2 | CTCTCCCACTTCGCTTGACTTC |
|  | p*AOX1*_TcPAND_in_RV | AGTAGCCTAGTAGAAGGAATTGG |
|  | 3'-HR_*RGI2*_out_FW | CTCGTCAGACATTAGTTCGC |
|  | 3’-HR_*RGI2*_seq2 | TATGACTACACTCAGTGTCG |
|  | 3'-HR_*RGI2*_out_RV | GGAGGTTCATTGGAATCTCG |
| PP7435_Chr2-0858 | 5'-HR_p*GAP*^UP^_out_FW | ATTGGAGCATCCTTGGATGG |
|  | 5'-HR_p*GAP*^UP^_seq2 | TATTATTGCCAGCGACGG |
|  | 3'-HR_p*GAP*^UP^_out_RV | AGTAATAATAGAGCAGAGCG |
| PP7435_Chr4-0130 | 5'-HR_p*AOX1*^UP^_out_FW | CTCTTTAATGAGGGGAATATCCTCAGG |
|  | 5'-HR_p*AOX1*^UP^_seq2 | GGGCATATGTGCTGGTGACAAAGG |
|  | 3'-HR_p*AOX1*^UP^_out_RV | GGGTTGTTGAGGTTGTTCTCACC |
|  | 3'-HR_p*AOX1*^UP^_seq1 | GTCATATTAGGTTCCAAGACAGCG |
| pGAP_gRNA_seq | | GCTGGAGAGCTTCTTCTACGGC |

1. Prielhofer R, Barrero JJ, Steuer S, Gassler T, Zahrl R, Baumann K, et al. Golden*Pi*CS: A Golden Gate-derived modular cloning system for applied synthetic biology in the yeast Pichia pastoris. BMC Syst Biol. 2017;11:123.
2. Gassler T, Heistinger L, Mattanovich D, Gasser B, Prielhofer R. CRISPR/Cas9-mediated homology-directed genome editing in Pichia pastoris. In: Gasser B, Mattanovich D, editors. Recombinant protein production in yeast. New York: Springer; 2019. p. 211-25.
